# Exploring the Therapeutic Effects of *Artemisia rupestris* L. Extract on Metabolic Dysfunction-Associated Fatty Liver Disease: Network Pharmacology and In Vitro Experiments

**DOI:** 10.3390/ph19091338

**Published:** 2026-08-24

**Authors:** Zheming Xiong, Yijie Su, Luping Shi, Shuyuan Ge, Ge Wang, Fangyu Li, Xu Liu, Jianguang Li, Zejiang Ma

**Affiliations:** 1 School of Traditional Chinese Medicine, Xinjiang Second Medical College, Karamay 834000, China; gesbear@163.com (Z.X.);; 2 School of Basic Medical Sciences, Xinjiang Second Medical College, Karamay 834000, China; suyijie@xjsmc.edu.cn (Y.S.);; 3 School of Pharmacy, Xinjiang Second Medical College, Karamay 834000, China

**Keywords:** *Artemisia rupestris* L. extract, metabolic dysfunction-associated fatty liver disease, UPLC-Q-TOF-MS, network pharmacology, hepatic steatosis

## Abstract

**Background**: Metabolic dysfunction-associated fatty liver disease (MAFLD) is a prevalent chronic liver disorder with limited pharmacological therapies, highlighting the need for novel multi-target agents from traditional medicinal plants. *Artemisia rupestris* L. has hepatoprotective effects, but its active constituents and mechanisms against MAFLD remain unexplored. **Methods**: The chemical profile of an ultrasonically-assisted 70% ethanol extract of *Artemisia rupestris* L. (ARE) was determined using UPLC-Q-TOF-MS. Network pharmacology, molecular docking, 100-ns molecular dynamics (MD) simulations, and in vitro experiments in oleic acid-induced HepG2 cells were integrated to identify active compounds, core targets, and pathways, and to validate lipid-lowering and hepatoprotective effects. **Results**: Seventy-eight compounds were identified, mainly flavonoids, phenolic acids, and terpenoids. Network analysis highlighted AKR1B10 and MAPK14 as core targets and isorupestonic acid and rupestonic acid as key components. MD simulations supported the stable binding of isorupestonic acid to AKR1B10 (RMSD ~1.6–3.2 Å), driven by van der Waals and electrostatic interactions. In vitro, ARE dose-dependently reduced intracellular triglycerides, total cholesterol, and LDL-C, decreased lipid droplets, and lowered AST/ALT levels, with a high-dose efficacy comparable to atorvastatin. **Conclusions**: Through in vitro experiments and network pharmacology analyses, this study preliminarily elucidated the beneficial effects of ARE on lipid deposition in hepatocytes and its potential mechanism, thereby providing an experimental basis and potential avenues for subsequent in vivo studies on pharmacodynamics, pharmacokinetics, and toxicology.

## 1. Introduction

Metabolic dysfunction-associated fatty liver disease (MAFLD) is a spectrum of liver diseases characterized by an imbalance in hepatic energy metabolism. This leads to the accumulation of lipids and triglycerides within hepatocytes, resulting in hepatocellular damage. MAFLD has become a leading cause of liver disease worldwide, affecting around 30 percent of the population [1]. MAFLD encompasses simple steatosis and metabolic dysfunction-associated steatohepatitis (MASH) [2]. Around 20 percent of patients with simple steatosis may progress to MASH, while approximately 30 percent of MASH patients progress to liver fibrosis [3]. The latter significantly increases the risk of cirrhosis and hepatocellular carcinoma. The main risk factors for MAFLD are insulin resistance, a high-fat and high-sugar diet, dyslipidemia, gut microbiota dysbiosis, and genetic factors [4]. Prior to the FDA’s approval of resmetirom and semaglutide in the past two years, there were no specific, effective drugs available for MAFLD [5]. Consequently, patients primarily relied on dietary adjustments and physical exercise to slow disease progression. However, given compliance issues, this approach is difficult to sustain [6]. Although these two drugs have been approved for use, their development costs are high and they are not yet widely used in clinical practice; there is therefore still a need to develop new, safe, effective, and low-cost drugs. In traditional Chinese medicine (TCM), MAFLD is regarded as ‘liver qi stagnation with spleen deficiency’ or ‘phlegm-dampness internal obstruction’. Due to their multi-component, multi-target nature, as well as their low toxicity and minimal side effects, TCM represents a valuable resource for the development of new drugs. A growing body of research indicates that the constituents of TCM have pharmacological properties such as lowering lipids and protecting the liver, offering a new perspective on the treatment of MAFLD. As a traditional medicinal plant, *Artemisia rupestris* L. has properties such as clearing damp-heat, promoting blood circulation, and detoxifying; these effects are highly consistent with the pathological basis of MAFLD in TCM.

*Artemisia rupestris* L. is a plant that belongs to the genus *Artemisia* in the Asteraceae family [7,8]. The medicinal material is derived from the plant’s aerial parts, which possess significant medicinal value and are documented in ancient Chinese medical texts such as the Compendium of Materia Medica, the Supplement to the Compendium of Materia Medica, and the Chinese Materia Medica [9]. The chemical composition of *Artemisia rupestris* L. is notable for the presence of various chemical compounds, including ketone acids, flavonoids, essential oils, alkaloids, amino acids, and lactones [10]. A plethora of clinical studies have demonstrated that the primary constituents of *Artemisia rupestris* L. possess a wide range of biological activities, including anti-allergic, stomach-soothing, and digestive-aiding properties, as well as detoxifying, antiviral, antibacterial, and liver-protective effects [8,11]. This extensive array of functionalities renders it a prevalent component in both traditional Chinese medicine and ethnic medicine. Nevertheless, the intricacies inherent in the chemical composition of traditional Chinese herbal medicines, in conjunction with the frequently uncharted therapeutic protein targets of these chemical compounds, impede the comprehension of TCM from a contemporary biomedical standpoint [12]. Consequently, a systematic analysis of the constituents in the medicinal parts of *Artemisia rupestris* L. is imperative for elucidating its biological activity and specific mechanisms of action. Adina et al. obtained an aqueous extract of *Artemisia rupestris* L. by subjecting it to three rounds of heating and reflux with 12-fold the volume of water, and identified the constituents using HPLC-Q-OT-qIT-MS. A total of 53 compounds were initially identified, primarily comprising flavonoids, sesquiterpenes, organic acids and their derivatives, polysaccharides, and coumarins [9]. Zhang Peijie et al. conducted a qualitative analysis of the chemical constituents of *Artemisia rupestris* L. using HPLC-IT-TOF-MS. A total of 124 compounds were detected in a 75% ethanol extract of *Artemisia rupestris* L., including chlorogenic acids, flavonoids, and sesquiterpenes [13]. Ultrasonic-assisted extraction, which enhances solvent penetration and active ingredient release, is known to improve extraction efficiency, yet it has not been applied to systematically investigate the chemical profile of *A. rupestris* for the study of MAFLD. Although prior research has explored the hepatoprotective effects of this plant, no study has systematically evaluated its pharmacological actions in MAFLD or identified the corresponding therapeutic targets of its constituents. Therefore, this study aims to combine ultrasound-assisted extraction with systematic chemical characterization and pharmacological research to investigate its medicinal value.

TCM is distinguished by its multi-component and multi-target nature, as well as complex mechanisms of action [14]. Consequently, it is extremely difficult to identify its active constituents and corresponding targets using traditional mechanistic analysis methods, which severely hampers modern medical research into TCM [12]. However, with the advent of modern bioinformatics, the nascent field of network pharmacology—which integrates existing disease target databases with proteomics and genomics data—is capable of addressing the challenges posed by multi-component, multi-target analysis. Moreover, given its alignment with the holistic philosophy of traditional TCM, it has been extensively adopted in TCM research. Consequently, by employing network pharmacology methods, it is possible to elucidate the target molecules corresponding to the constituents of *Artemisia rupestris* L. extract. This builds upon the identification of constituents and the determination of pharmacological effects [15,16]. This, in turn, helps us better understand its potential pharmacological mechanisms for treating MAFLD.

Consequently, this study aims to characterize the chemical composition of a 70% ethanol extract of *Artemisia rupestris* L. (ARE) using an UPLC-Q-TOF-MS system, evaluate its effect on improving oleic acid (OA)-induced lipid accumulation in HepG2 cells, and integrate network pharmacology techniques such as molecular dynamics simulations and enrichment analysis, to preliminarily elucidate the novel pharmacological effects of ARE against MAFLD, predict potential bioactive components and mechanisms of action, and provide a research foundation for the development of new therapeutic applications and the high-value utilization of *Artemisia rupestris* L.

## 2. Results

### 2.1. Analysis of ARE Components

**Figure 1:**
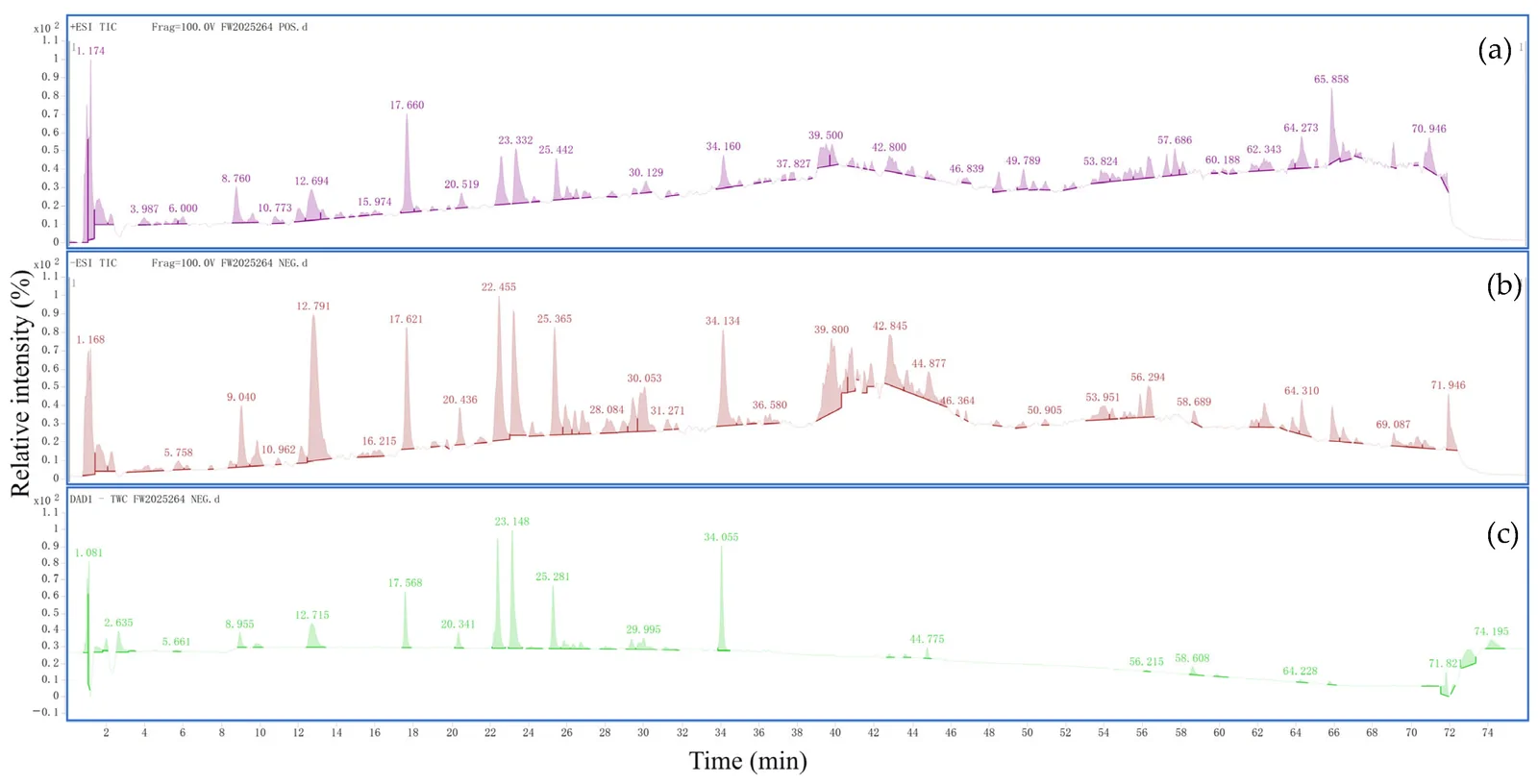
Total ion chromatogram of ARE. (**a**) Positive ion mode; (**b**) negative ion mode; (**c**) full-wavelength UV absorption spectrum.

The chemical composition of ARE was analyzed using UPLC-Q-TOF-MS (Figure 1). A total of 78 compounds were tentatively annotated by referencing the relevant literature and databases. Among them, the three most abundant categories were 24 flavonoids, 12 organic acids, and 11 terpenoids. The detailed results are presented in Table 1.

### 2.2. Target Identification

#### 2.2.1. Identification of Disease Targets

Differential expression analysis was first performed on four datasets. A total of 7760 differentially expressed genes (DEGs) were obtained from GSE89632, 1225 from GSE160016, 6859 from GSE126848, and 55 from GSE151158. To avoid missing potential targets, the union of DEGs from the four datasets was taken, yielding 11,783 disease-associated genes. Subsequently, these 11,783 genes were annotated using the GSE135251 dataset, resulting in 10,525 successfully annotated genes, whose expression profiles were then extracted. The details of the GEO datasets are shown in Table A1.

**Figure A1:**
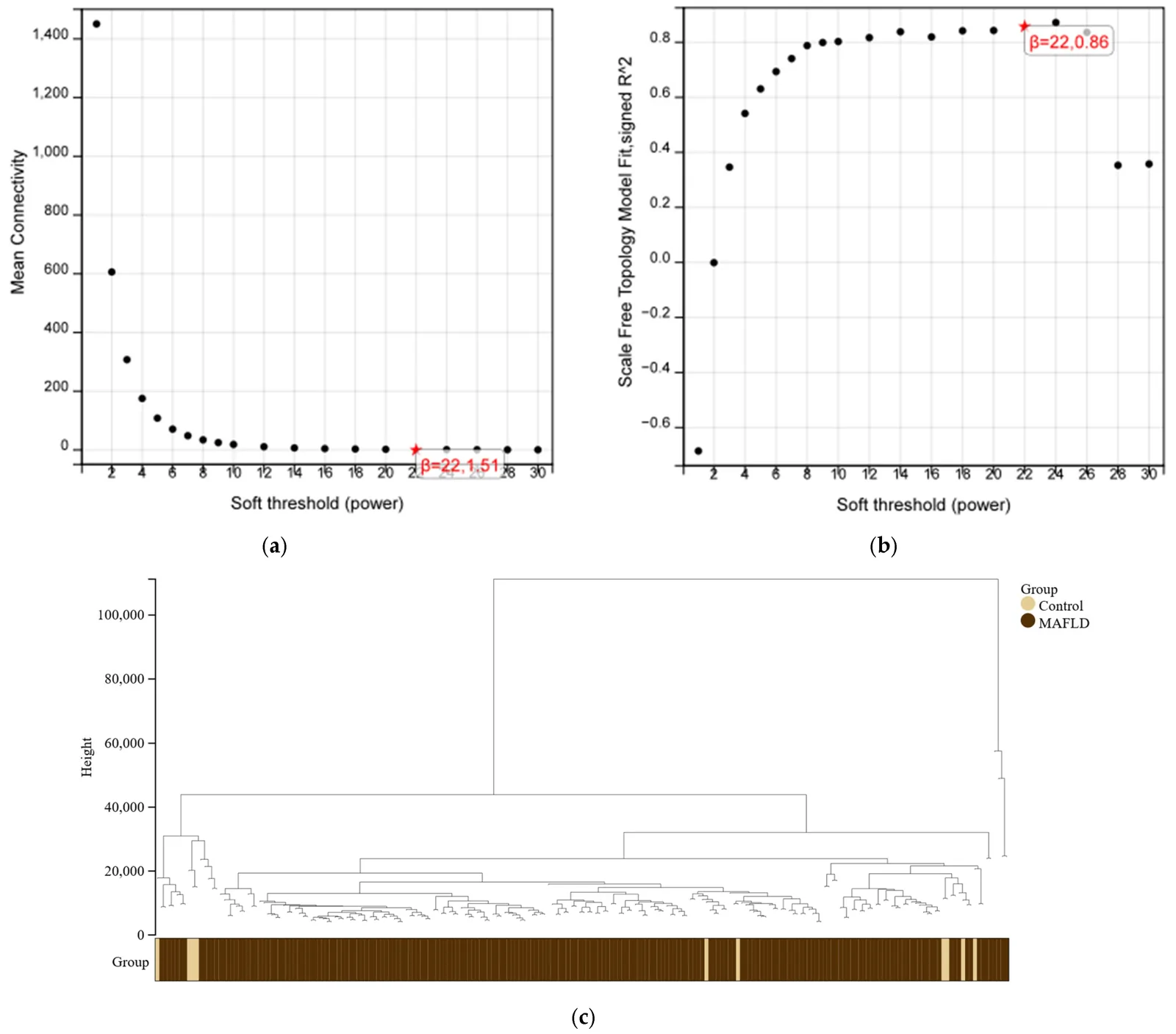
Soft threshold screening and sample clustering diagram. (**a**) Scale-free model; (**b**) average connectivity analysis; (**c**) sample clustering.

**Figure A2:**
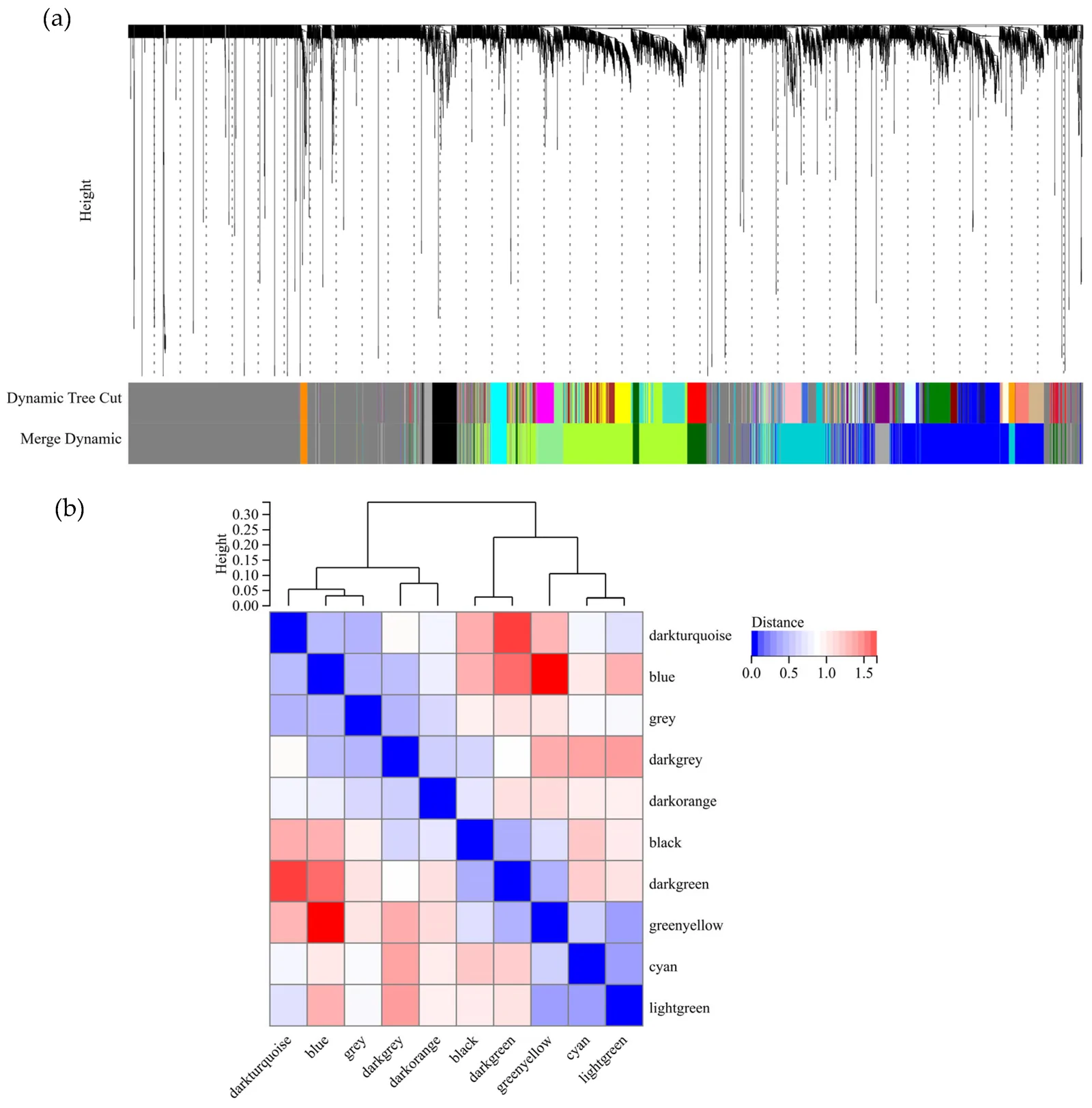
Gene clustering and module correlation tree. (**a**) Gene cluster tree; (**b**) module correlation analysis.

WGCNA was further performed on the GSE135251 dataset to identify genes associated with the MAFLD phenotype. The soft threshold was set to 22 (Figure A1a,b), indicating that after raising the adjacency matrix to the 22nd power, the connections among genes in the network more closely followed a scale-free network distribution, thereby enhancing the biological relevance of the expression profile analysis. All retained genes were classified into 10 distinct modules (Figure A1c), with the number of genes in each module summarized in Table A2. These modules were broadly grouped into two major clusters: one comprising dark turquoise, blue, grey, dark grey, and dark orange modules, and the other consisting of the remaining five modules (Figure A2).

**Figure 2:**
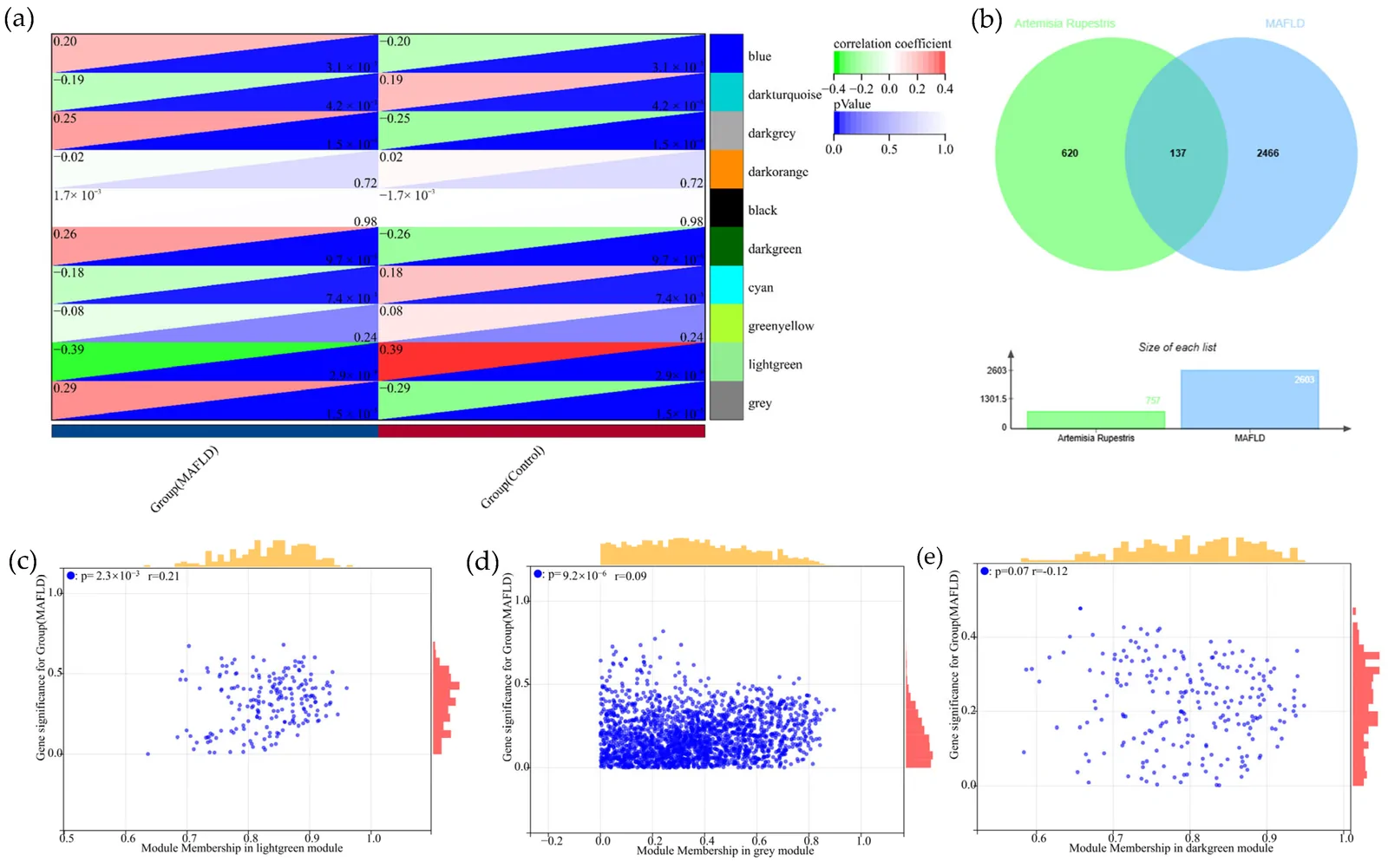
Correlation analysis between modules and clinical features. (**a**) Correlation between modules and clinical features. (**b**) Correlation between light green modules and MAFLD. (**c**) Correlation between grey modules and MAFLD. (**d**) Correlation between dark green modules and MAFLD. (**e**) Intersection targets.

Phenotype correlation analysis revealed that the dark orange and black modules exhibited the lowest correlation with the phenotype. Aside from these, all other modules showed some degree of correlation with the two phenotypes (MAFLD patients vs. healthy controls) (Figure 2a and Table 2). Among them, the light green module displayed the highest correlation with the phenotype (R = −0.39, *p* = 2.9 × 10^−9^; Figure 2b), followed by the grey module (R = 0.26, *p* = 1.5 × 10^−5^; Figure 2c) and the dark green module (R = 0.26, *p* = 9.7 × 10^−5^; Figure 2d). These findings suggest that genes within these three modules are the most likely key drivers of MAFLD initiation and progression. Finally, these three modules were merged, yielding 2603 disease targets closely associated with MAFLD.

#### 2.2.2. Acquisition of Targets of ARE

We retrieved the structural formulas of the 78 previously identified components from PubChem. Components for which targets could not be predicted or whose target confidence was zero were excluded. After deduplication, the remaining 74 components were found to share a total of 757 targets. Finally, the intersection of disease targets and drug targets was taken, yielding 137 common targets (Figure 2e), suggesting that ARE may exert its therapeutic effects against MAFLD through these targets.

### 2.3. Protein–Protein Interaction (PPI) Analysis and Enrichment Analysis

**Figure 3:**
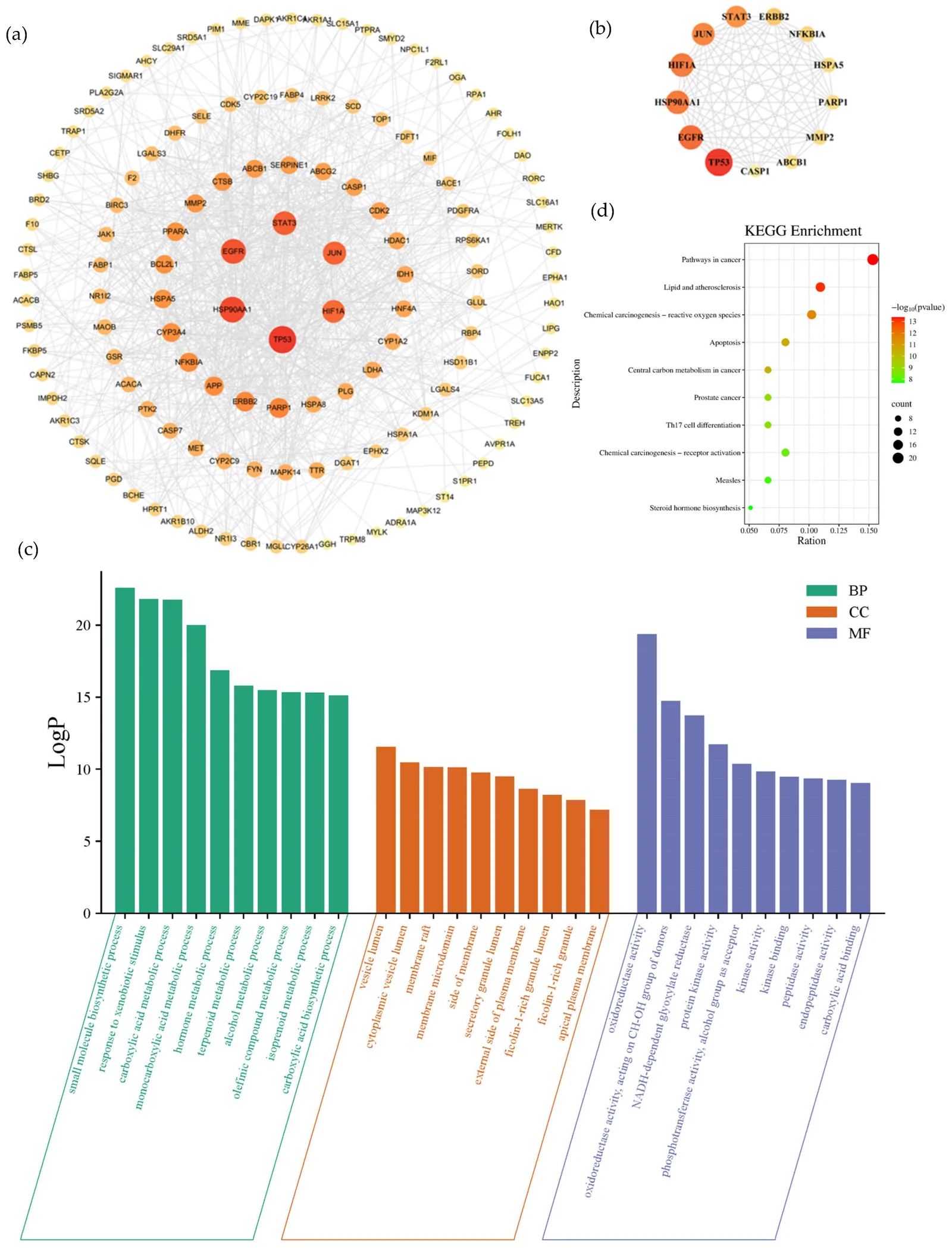
Construction of the PPI network and enrichment analysis. (**a**) PPI; (**b**) hub proteins; (**c**) GO; (**d**) KEGG.

A total of 137 overlapping targets were submitted to the STRING database to construct a protein–protein interaction (PPI) network, which comprised 131 nodes and 879 edges (Figure 3a). Using the CytoNCA plugin in Cytoscape (version 3.10.4, Cytoscape Consortium, San Diego, CA, USA) with filtering criteria (degree centrality ≥ 16, closeness centrality ≥ 0.633, betweenness centrality ≥ 8.113, eigenvector centrality ≥ 0.147, LAC ≥ 10.706, and network structural index ≥ 12.394), 13 core targets were obtained (Figure 3b). Among them, the top 10 ranked by degree were TP53, EGFR, HSP90AA1, HIF1A, JUN, STAT3, ERBB2, NFKBIA, HSPA5, and PARP1, suggesting that *Artemisia rupestris* L. may exert anti-MAFLD effects through multiple targets.

GO enrichment analysis identified 1078 terms (865 BP, 79 CC, 143 MF). The top 10 terms from each category are shown in Figure 3c. The main BP terms included small molecule biosynthetic process, response to xenobiotic stimulus, carboxylic acid metabolic process, monocarboxylic acid metabolic process, hormone metabolic process, terpenoid metabolic process, alcohol metabolic process, olefinic compound metabolic process, isoprenoid metabolic process, and carboxylic acid biosynthetic process. The CC terms primarily involved vesicle lumen, cytoplasmic vesicle lumen, membrane raft, membrane microdomain, side of membrane, secretory granule lumen, external side of plasma membrane, ficolin-1-rich granule lumen, ficolin-1-rich granule, and apical plasma membrane. The MF terms included oxidoreductase activity, oxidoreductase activity acting on the CH-OH group of donors, NADH-dependent glyoxylate reductase activity, protein kinase activity, phosphotransferase activity (alcohol group as acceptor), kinase activity, kinase binding, peptidase activity, endopeptidase activity, and carboxylic acid binding.

KEGG pathway enrichment analysis yielded 145 pathways. The top 10 significant pathways (by *p*-value) are presented in Figure 3d, including pathways in cancer, lipid and atherosclerosis, chemical carcinogenesis—reactive oxygen species, apoptosis, central carbon metabolism in cancer, prostate cancer, Th17 cell differentiation, chemical carcinogenesis—receptor activation, measles, and steroid hormone biosynthesis.

### 2.4. Construction of the Component–Target–Pathway Network

**Figure 4:**
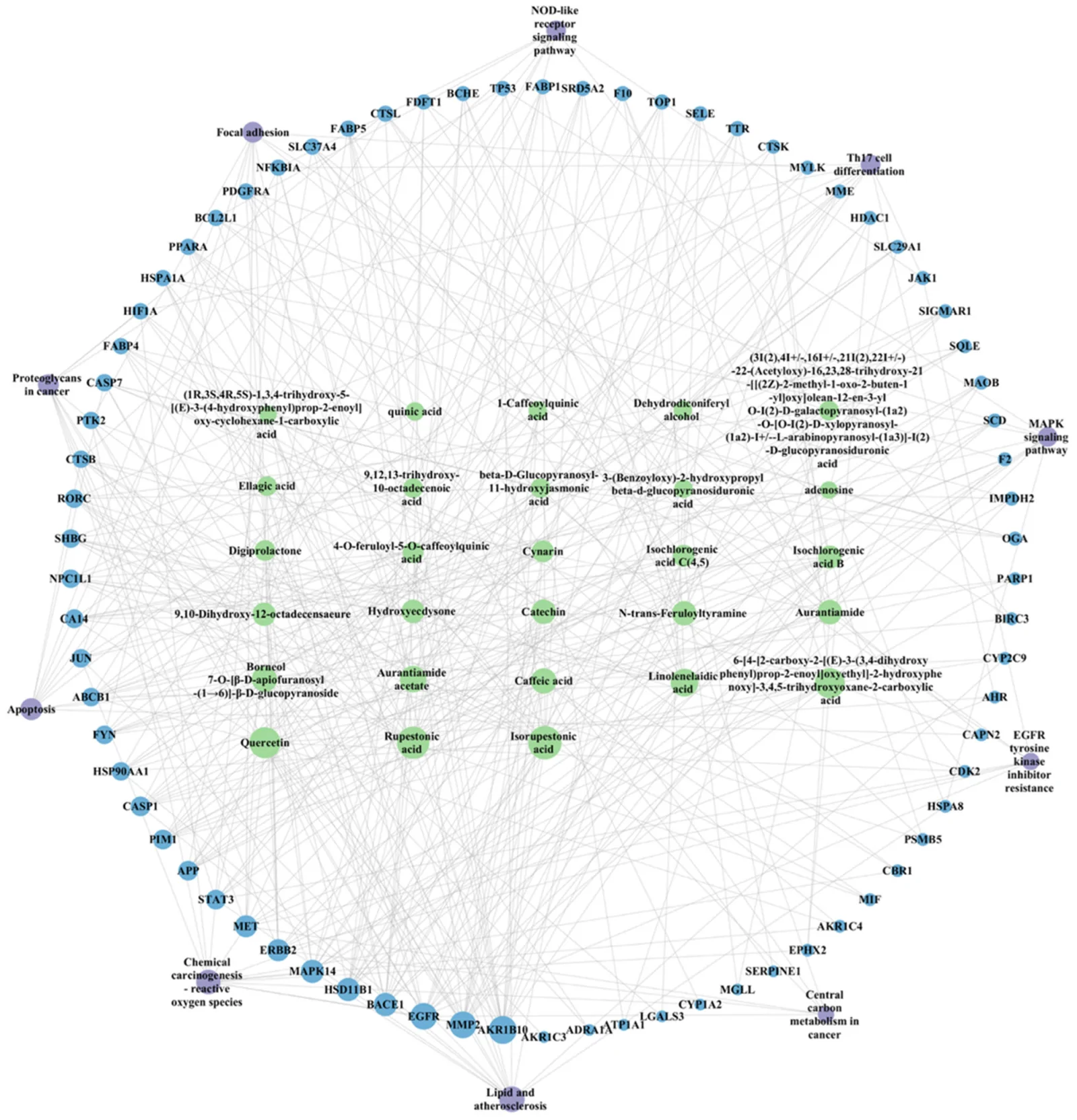
Component–target–pathway network. In the network, green squares represent active components, blue circles represent potential targets, and purple triangles represent pathways. The size of each node is proportional to its degree value, with larger nodes indicating higher connectivity. Edges represent interactions between components and targets or between targets and pathways. The network illustrates the multi-component, multi-target, and multi-pathway mechanism of ARE against MAFLD.

A component–target–pathway network was constructed using Cytoscape (version 3.10.4, Cytoscape Consortium, San Diego, CA, USA), as shown in Figure 4. In this network, squares, circles, and triangles represent active components, targets, and pathways, respectively. After removing low-degree peripheral nodes, the core network comprised 112 nodes and 433 edges (Figure 4). Topological analysis identified the top 10 core targets by degree value: AKR1B10, MMP2, EGFR, BACE1, HSD11B1, MAPK14, ERBB2, MET, STAT3, and APP. The top 10 active components predicted by network pharmacology were isorupestonic acid, rupestonic acid, quercetin, 6-[4-[2-carboxy-2-[(E)-3-(3,4-dihydroxyphenyl)prop-2-enoyl]oxyethyl]-2-hydroxyphenoxy]-3,4,5-trihydroxyoxane-2-carboxylic acid, linolenelaidic acid, caffeic acid, aurantiamide acetate, borneol 7-O-[β-D-apiofuranosyl-(1→6)]-β-D-glucopyranoside (ophiopogonin D), aurantiamide, and N-trans-feruloyltyramine. The top 10 targets, active components, and pathways (ranked by degree separately) predicted by network pharmacology are summarized in Table A3.

### 2.5. Molecular Docking Results

**Figure 5:**
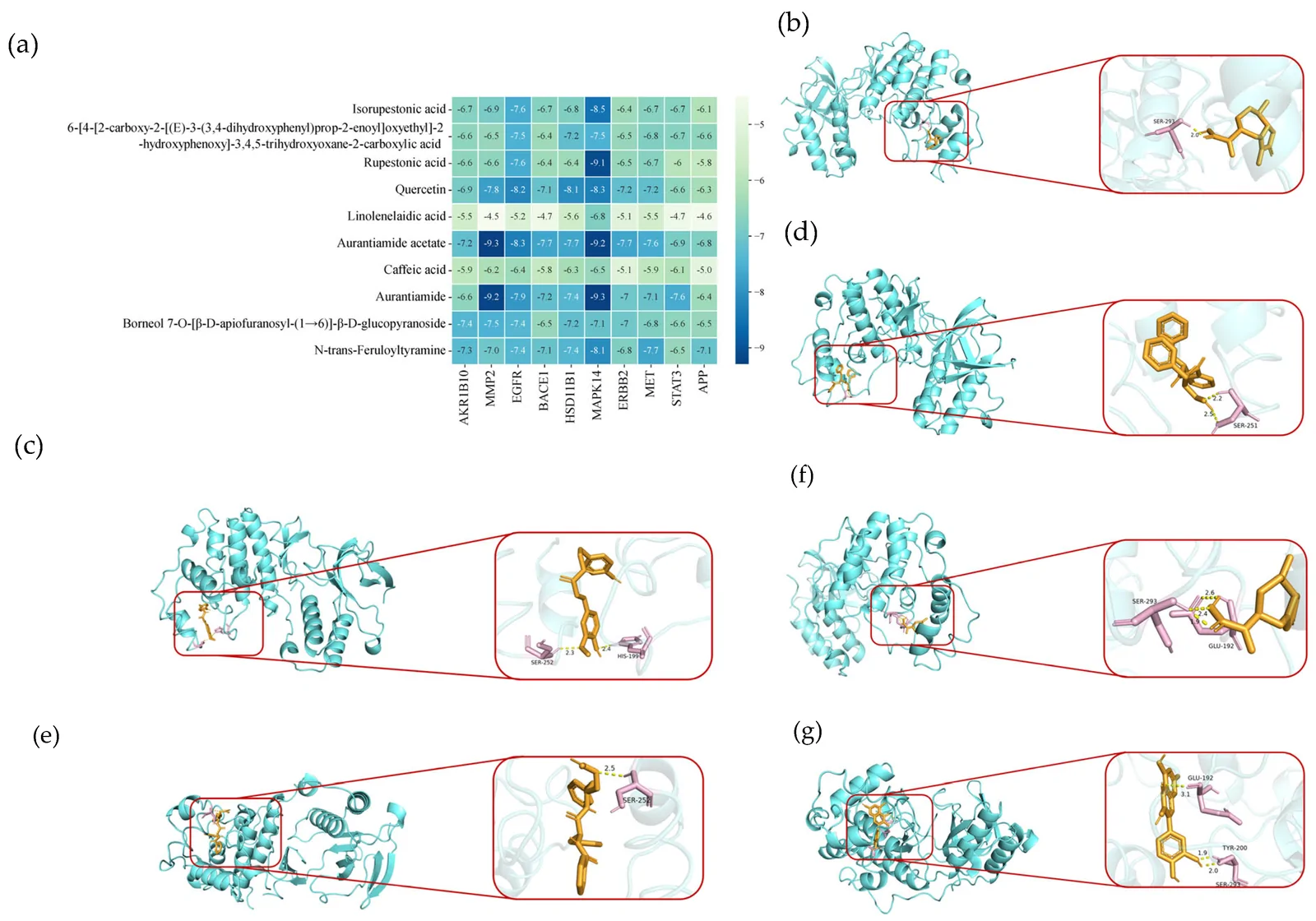
Molecular docking results. (**a**) Binding energy heatmap of the top 10 components against the top 10 targets. (**b**–**g**) Representative docking poses of compounds with binding energies less than −8 kcal/mol to MAPK14. (**b**) Rupestonic acid; (**c**) N-trans-feruloyltyramine; (**d**) aurantiamide; (**e**) aurantiamide acetate; (**f**) isorupestonic acid; (**g**) quercetin.

Based on the degree values in the component–target–pathway network, the top 10 active components and top 10 targets were selected for molecular docking analysis. As shown in Figure 5a, the binding energies of most component–target pairs were below −5 kcal/mol, indicating good binding potential. Notably, the binding energies of all tested components with MAPK14 were below −7 kcal/mol. Among them, rupestonic acid, aurantiamide acetate, and aurantiamide exhibited binding energies of −9.1, −9.2, and −9.3 kcal/mol, respectively, suggesting that these components may directly act on MAPK14 (Figure 5b–f).

### 2.6. Molecular Dynamics Simulation Results

Although MAPK14 exhibited higher binding affinity in molecular docking analyses, AKR1B10 was prioritized for molecular dynamics simulation due to its ranking as the most central node (degree = 16; see Table A3 and Figure 4) within the component–target–pathway network. This indicates a more central regulatory role in lipid metabolism. In contrast, MAPK14 ranked fourth with a degree value of 12 (Table A3). Moreover, AKR1B10 had been established as a key biomarker and core enzyme in glycerol lipid metabolism during MAFLD development and progression.

**Figure A3:**
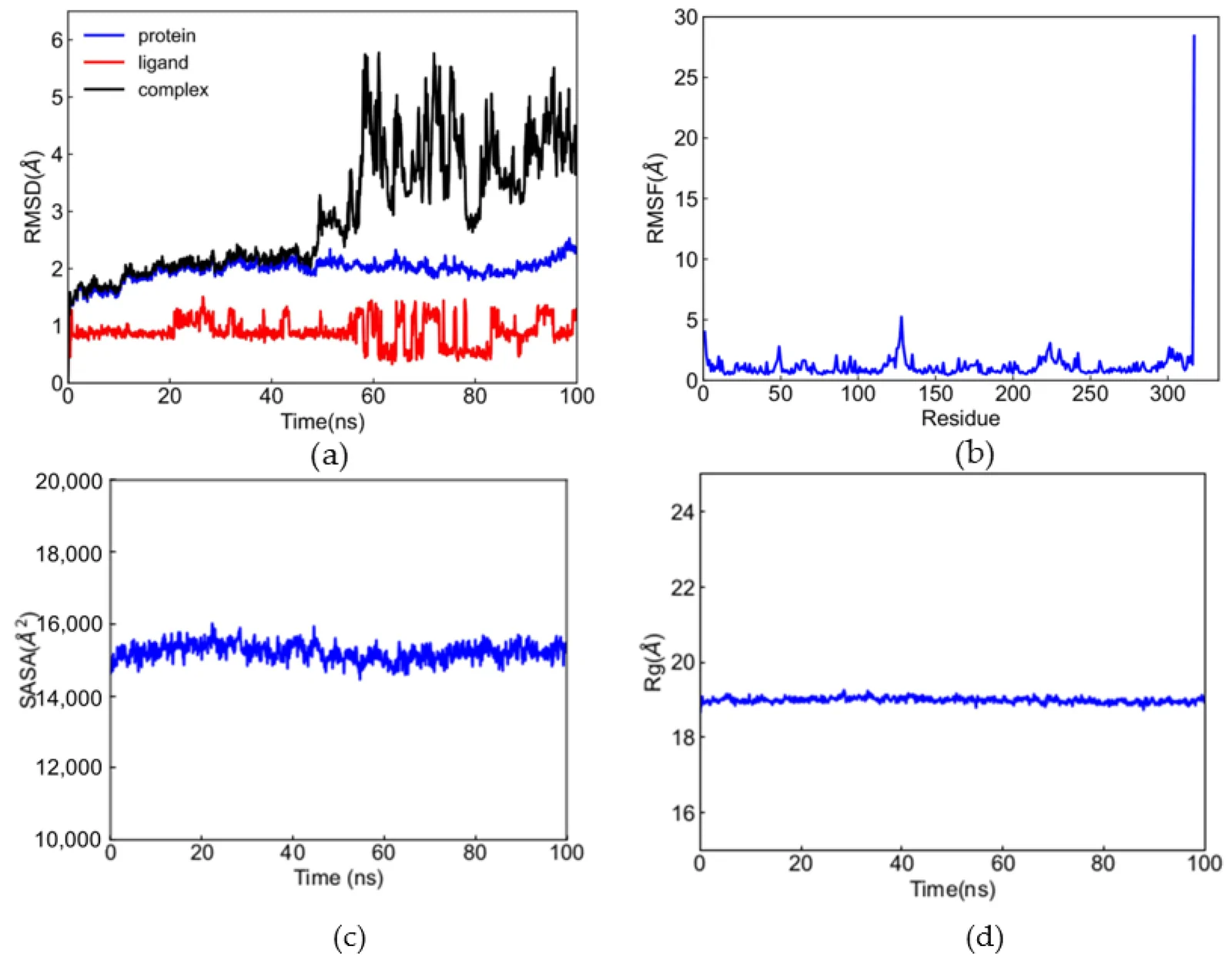
Molecular dynamics simulation results for the rupestonic acid–AKR1B10 complex over a 100 ns time span. (**a**) Root mean square deviation (RMSD) as a function of time. (**b**) Radius of gyration (Rg) as a function of time. (**c**) Solvent-accessible surface area (SASA) as a function of time. (**d**) Root mean square fluctuation (RMSF) for each residue.

The two characteristic components, rupestonic acid and isorupestonic acid, exhibited binding energies of −6.6 and −6.7 kcal/mol with AKR1B10, respectively, both of which remained below the generally accepted activity threshold (−5 kcal/mol), suggesting stable binding potential. Considering the functional importance of the target in the disease, its topological status in the network, and its effective binding with the characteristic components, we selected AKR1B10 for subsequent molecular dynamics simulations to validate its binding stability. During the 100 ns simulation, rupestonic acid dissociated from the binding site at 50 ns (Figure A3). In contrast, isorupestonic acid remained stably bound throughout the simulation.

**Figure 6:**
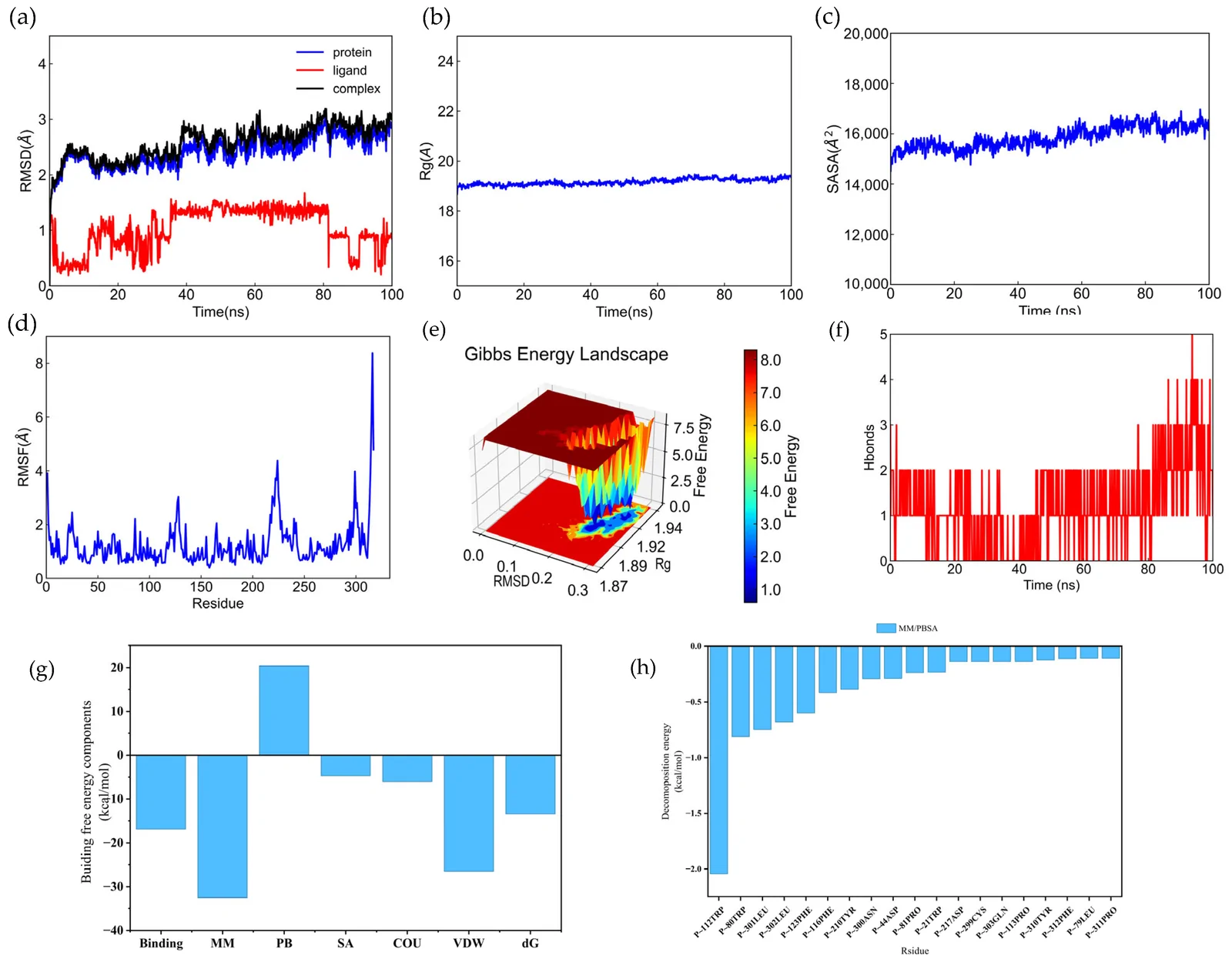
Molecular dynamics simulation results of the isorupestonic acid–AKR1B10 complex over 100 ns. (**a**) Root-mean-square deviation (RMSD) as a function of time, reflecting the overall conformational stability of the complex during the simulation. (**b**) Radius of gyration (Rg) over time, evaluating the compactness of the protein–ligand complex structure. (**c**) Solvent-accessible surface area (SASA) over time, illustrating the effect of binding on protein surface exposure to solvent. (**d**) Root-mean-square fluctuation (RMSF) per residue, showing the local flexibility of individual amino acid residues during the simulation. (**e**) Free energy landscape (FEL) plotted as a function of RMSD and Rg, identifying the most stable conformational region (low-energy valley) of the system. (**f**) Number of hydrogen bonds over time, reflecting the dynamic evolution and stability of hydrogen-bonding interactions. (**g**) Total binding free energy calculated by the MM/PBSA method was –13.368 kcal/mol; the negative value indicates a stable binding process with strong affinity. (**h**) Per-residue contribution to the binding free energy (in kcal/mol), showing that TRP112 exhibits a substantial contribution (absolute value > 1.0 kcal/mol), suggesting a key role of this residue in stabilizing the ligand-binding conformation.

As shown in Figure 6a, the RMSD of the isorupestonic acid–AKR1B10 complex rapidly increased during the initial 20 ns and then plateaued, fluctuating within a stable range of approximately 1.6–3.2 Å for the remaining 80 ns of the 100 ns simulation, indicating that the system had reached conformational equilibrium and the complex remained stable throughout the production phase. The radius of gyration (Rg) fluctuated within 16–24 Å, suggesting no significant change in protein compactness (Figure 6b). The solvent-accessible surface area (SASA) varied between 10,000 and 20,000 Å^2^, without abnormal exposure or aggregation (Figure 6c). RMSF values for most residues ranged from 2 to 4 Å; higher fluctuations were observed in loop regions, whereas key binding-site residues remained relatively stable (Figure 6d). The number of hydrogen bonds between the ligand and protein fluctuated mainly between 0 and 4, with an average of 1–2, indicating few but stable hydrogen-bonding interactions (Figure 6f).

The 2D/3D free energy landscape showed a low-energy region (<2 kcal/mol) at RMSD 1.87–1.94 Å and Rg 1.87–1.94 Å, representing a dominant stable binding conformation (Figure 6d). Hydrogen bond analysis using the GROMACS hbond tool revealed that the isorupestonic acid–AKR1B10 complex maintained 0–4 hydrogen bonds throughout the 100 ns simulation, with an average of 1–2 interactions. The primary hydrogen-bonding residues were LEU302 (occupancy: 35.1%), LEU301 (23.4%), HSD111 (15.3%), ASN161 (14.2%), and ARG125 (13.1%), which together accounted for the majority of the observed hydrogen-bonding events. Notably, the hydrogen bond network underwent dynamic reorganization during the simulation: in the early phase (0–50 ns), TYR49, TRP21, ASN161, and ARG125 were the major contributors, whereas in the later phase (50–100 ns), LEU301, LEU302, and HSD111 became predominant. The MM/PBSA binding free energy of the isorupestonic acid–AKR1B10 complex was calculated as −13.368 kcal/mol, with a standard deviation of ±2.10 kcal/mol over the sampled trajectory. The detailed energy components were as follows: MM −32.52 ± 2.70 kcal/mol, PB 20.33 ± 2.48 kcal/mol, SA −4.69 ± 0.15 kcal/mol, COU −6.03 ± 2.44 kcal/mol, and VDW −26.49 ± 1.57 kcal/mol. The relatively narrow standard deviation indicates consistently favorable binding throughout the sampled period, confirming that the estimated affinity is not dependent on a limited number of rare conformations (Figure 6g). Per residue energy decomposition analysis revealed that TRP112 exhibited a dominant contribution to ligand binding, with a value of −2.04 kcal/mol—substantially higher than all other residues (the second highest contribution was −0.75 kcal/mol for LEU301). This marked difference confirms the specific and predominant role of TRP112 in stabilizing the isorupestonic acid–AKR1B10 complex (Figure 6h).

In summary, the MD simulation system reached good equilibrium within 100 ns, with the predicted stable binding of isorupestonic acid with AKR1B10. The binding was driven primarily by van der Waals and electrostatic interactions, with key contributions from TRP, LEU, PHE, and TYR residues. The complex maintained a compact structure with reasonable flexibility, and the free energy landscape revealed a single stable conformational basin, supporting a reliable binding mode.

### 2.7. Results of In Vitro Inhibition of Lipid Deposition in Hepatocytes by ARE

**Figure 7:**
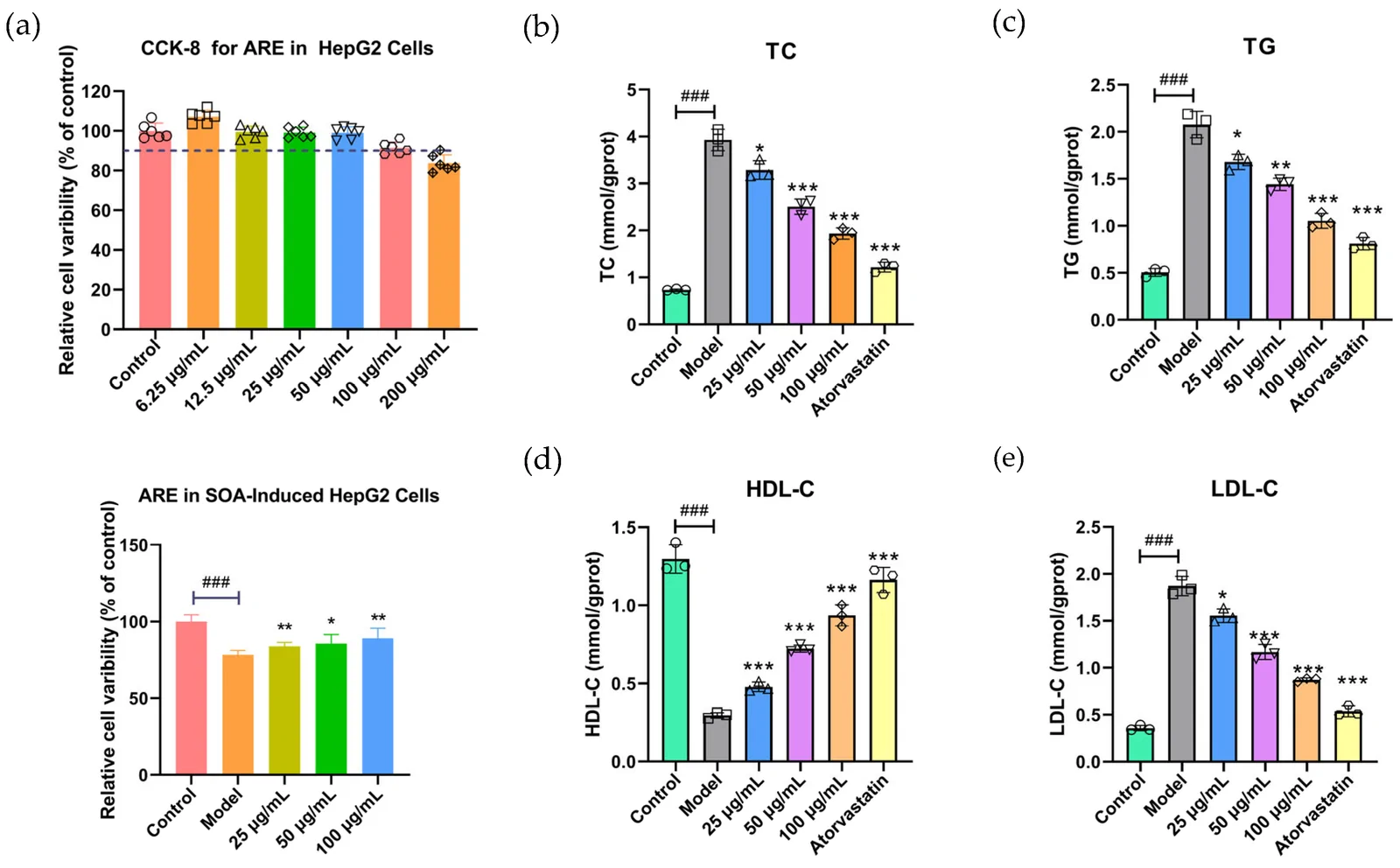
Results of the in vitro lipid-lowering trial of ARE. (**a**) Cell viability of various ARE concentrations both in HepG2 cells and SOA-induced HepG2 cells, The dashed lines indicate 90% cell viability; (**b**) TC concentration; (**c**) TG concentration; (**d**) LDL-C concentration; (**e**) HDL-C concentration; n = 3, Model vs. Control, ^###^ *p* < 0.001; other groups vs. Model, * *p* < 0.05, ** *p* < 0.01, *** *p* < 0.001.

**Figure 8:**
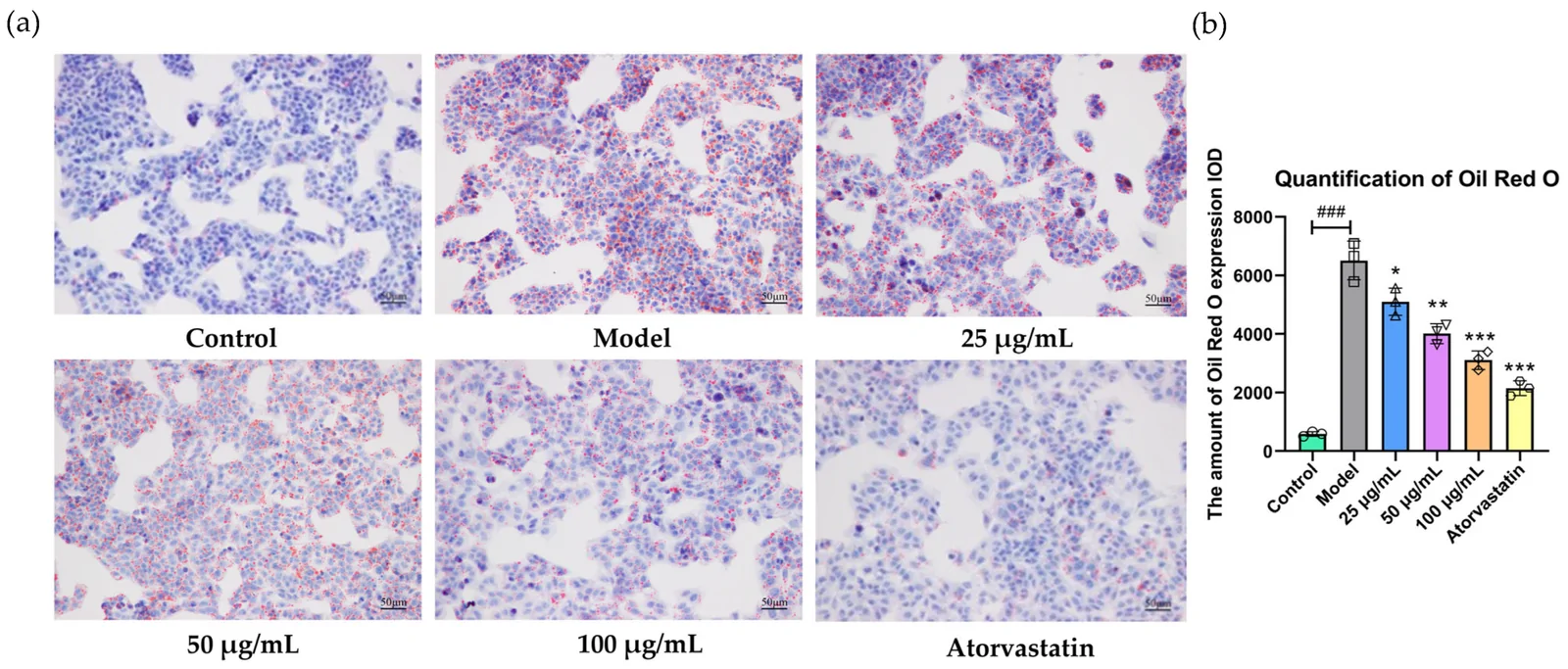
Results of Oil Red O staining. (**a**) Representative images of Oil Red O staining for each group; (**b**) quantitative results of Oil Red O staining; n = 3, Model vs. Control, ^###^ *p* < 0.001; other groups vs. Model, * *p* < 0.05, ** *p* < 0.01, *** *p* < 0.001.

To evaluate the anti-MAFLD activity of ARE, we established an in vitro model of hepatic lipid accumulation and assessed the pharmacological effects of ARE. CCK-8 results indicated that within an ARE concentration range of 6.25–100 µg/mL, the survival rate of HepG2 cells remained above 90%. In an oleic acid-induced model of hepatic lipid accumulation, ARE was able to restore the survival rate that had been reduced by the model (Figure 7a). On this basis, we further selected ARE concentrations of 25, 50, and 100 µg/mL as the low, medium, and high-dose treatment groups, whilst using atorvastatin (10 μM) as the positive control for subsequent experiments. Compared with the model group (induced with 0.5 mM sodium oleate for 24 h), the ARE-treated groups exhibited dose-dependent reductions in TG, TC, and LDL-C levels within hepatocytes, alongside a dose-dependent increase in HDL-C; moreover, the high-dose ARE group demonstrated efficacy comparable to that of atorvastatin (Figure 7b–e). The lipid-lowering effect of ARE was further validated by Oil Red O staining; the results indicated that ARE treatment could dose-dependently reduce model-induced lipid droplet accumulation in hepatocytes (Figure 8a,b). These findings collectively suggested that ARE exhibited a significant lipid-lowering effect in an in vitro hepatocyte lipid deposition model.

### 2.8. Results on the In Vitro Hepatoprotective Effects of ARE

**Figure 9:**
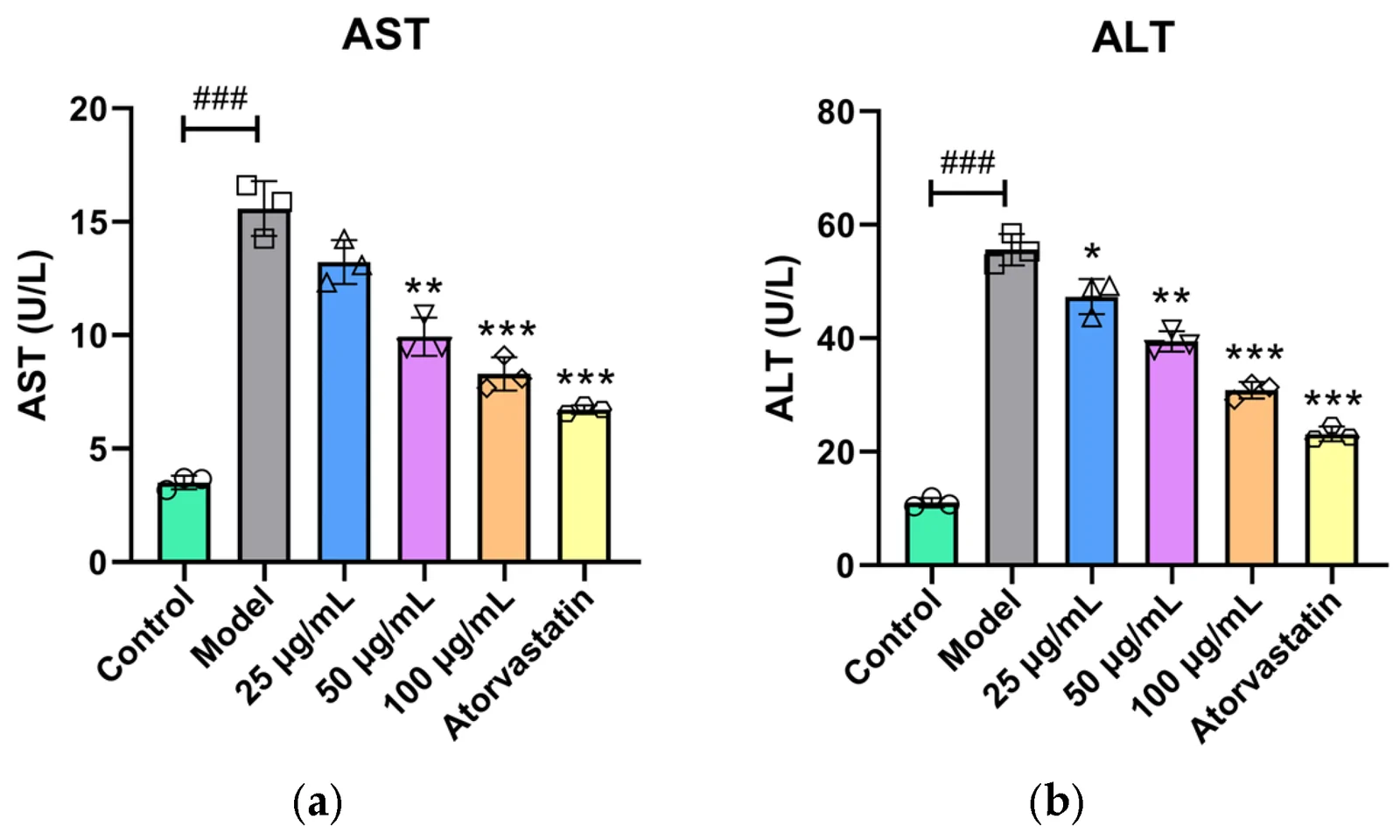
Results of tests for markers of hepatocyte damage. (**a**) AST concentration in cell culture supernatant; (**b**) ALT concentration in cell culture supernatant; n = 3, Model vs. Control, ^###^ *p* < 0.001; other groups vs. Model, * *p* < 0.05, ** *p* < 0.01, *** *p* < 0.001.

To further evaluate the hepatoprotective effects of ARE, we measured the liver damage markers AST and ALT in hepatocyte culture medium. The results are shown in Figure 9. Compared with the model group, ARE was able to reduce the secretion of AST and ALT by hepatocytes in a dose-dependent manner, suggesting that ARE possesses good hepatoprotective effects.

### 2.9. ARE Reduced the Levels of AKR1B10 Protein and mRNA in SOA-Induced HepG2 Cells

**Figure 10:**
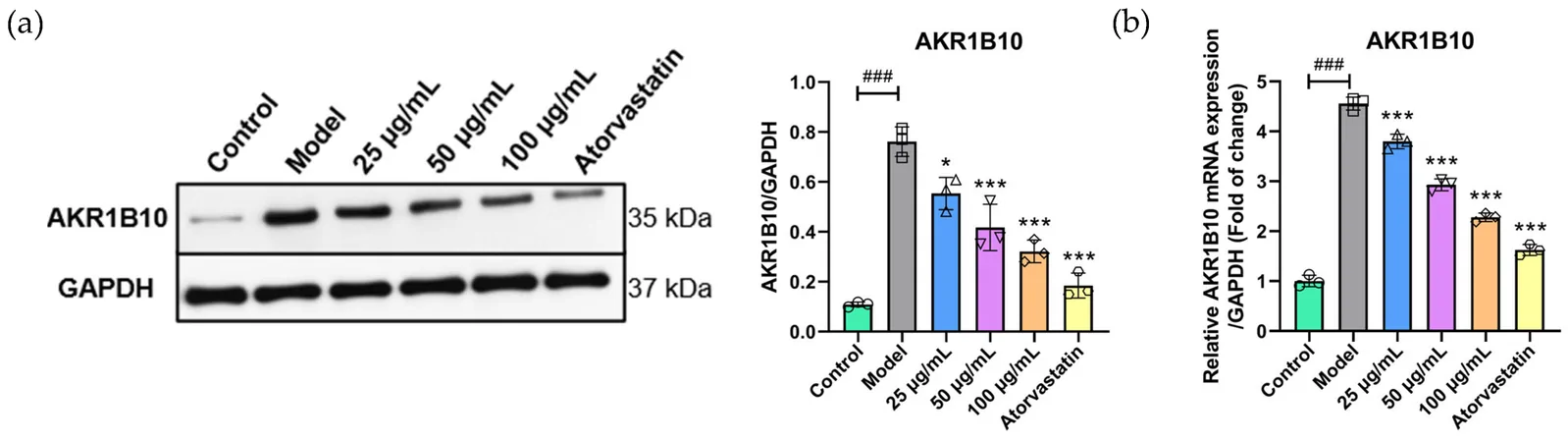
Results of tests for the levels of AKR1B10 protein and mRNA in SOA-induced hepG2 cells. (**a**) Representative immunoblots and quantification of AKR1B10 expression treatment with ARE or Atorvastatin (n = 3). (**b**) Relative gene expression levels of AKR1B10 treatment with ARE or Atorvastatin (n = 3); Model vs. Control, ^###^ *p* < 0.001; other groups vs. Model, * *p* < 0.05, *** *p* < 0.001.

Based on preliminary network pharmacology screening, which identified AKR1B10 as the core target, we conducted Western blot and RT-qPCR experiments to verify whether ARE can regulate its levels. As illustrated in Figure 10, ARE was found to significantly downregulate SOA-induced increases in AKR1B10 protein and mRNA levels. This suggests that AKR1B10 is one of the key targets of ARE’s lipid-lowering effect.

## 3. Discussion

In recent years, the prevalence of MAFLD has continued to rise worldwide. Current estimates suggest that approximately one-third of the global adult population is affected by MAFLD, making it the most prevalent chronic liver disease globally [17]. However, to date, only resmetirom and semaglutide—approved by the FDA in 2024 and 2025, respectively—are available on the market, which cannot fully meet the therapeutic needs for MAFLD [17]. TCM offers advantages such as low toxicity and multi-target action. Existing research indicates that various Chinese herbal medicines show potential in the treatment of MAFLD [14]; for example, the total extract of Rhododendron leaves has been shown to alleviate MAFLD by regulating the PPAR and TNF signaling pathways [18], whilst Eucommia has been demonstrated to alleviate MAFLD via the GPR43/GLP-1 receptors [19]. Our study employed UPLC-Q-TOF-MS technology to identify the major constituents of a 70% ethanol extract of *Artemisia rupestris* L. and screened for the active components and corresponding targets of ARE against MAFLD using an integrated network pharmacology approach combined with kinetic modeling. Furthermore, an in vitro model of hepatic lipid deposition was established to preliminarily validate the lipid-lowering efficacy of ARE, thereby laying a research foundation for elucidating its pharmacological mechanisms against MAFLD and for new drug development.

A total of 78 compounds were identified in ARE using UPLC-Q-TOF-MS technology. All compounds were identified by comparing the obtained MS/MS spectra with those in the PCDL and SIRIUS (version 5.8.5) databases, rather than by confirming them against standard samples. In future, reference standards will still be necessary to increase confidence in compound identification. To avoid overlooking potential active compounds, some compound annotations with lower confidence levels (compounds **25** and **42**) were still retained in the preliminary screening, of which 24 were flavonoids and their glycosides (compounds **11**, **16**, **21**, **22**, **32**–**35**, **38**–**41**, **45**, **46**, **49**–**53**, **55**, **57**, **62**, **63**, and **65**). Flavonoids have been shown to have hypoglycemic, lipid-regulating, and hepatoprotective properties [20]. For instance, quercetin (**63**), a dietary flavonoid found in many traditional Chinese medicinal products, has antioxidant and blood glucose-regulating properties and has been shown to lower lipids in obese mice [21]. Rutin (**38**), another natural flavonoid, improves lipid metabolic dysfunction in diabetic non-alcoholic steatohepatitis (NASH) via the AMPK/SREBP1 pathway [22]. A total of 21 phenolic acids and chlorogenic acid esters (**1**, **4**, **7**, **9**, **12**, **15**, **17**, **18**, **19**, **24**, **27**–**29**, **37**, **44**, **47**, **54**, **56**, **58**, **59**, and **61**) were also identified. This class of compounds is widely distributed in medicinal and edible plants, possessing a variety of pharmacological activities such as anti-inflammatory and antioxidant effects, as well as alleviating obesity and diabetes [23]. Their mechanisms are associated with regulating glucose and lipid metabolism and modulating the gut microbiota. Seven terpenoids and steroids were also identified (**8**, **30**, **31**, **42**, **64**, **70**, and **78**). Rupestonic acid (**30**) and isorupestonic acid (**70**) are characteristic compounds of Artemisia annua and have the potential to serve as *Artemisia rupestris* L. quality markers [9]. Both compounds ranked among the top 10 in the compound–target–pathway network. Studies indicate that rupestonic acid exhibits antiviral, anti-tumor, and anti-pulmonary fibrosis activities [11,24,25]. Our results showed that these two components were predicted by network pharmacology analysis to be key contributors to ARE’s anti-MAFLD activity. This provided direction for future efforts to identify ARE’s active moieties through the isolation of individual compounds and independent pharmacodynamic experiments. Additionally, eight aliphatic organic acids/fatty acid derivatives (**2**, **20**, **23**, **66**, and **73**–**76**), nine nitrogen-containing compounds (**3**, **5**, **25**, **60**, **68**, **69**, **71**, **72**, and **77**) and five phenylpropanoids/coumarins/lignans (**6**, **13**, **14**, **36**, and **43**) were identified, as well as three compounds from other categories (**26**, **48**, and **67**). Overall, our findings suggest that ARE possesses lipid-lowering effects, which may be attributable to the synergistic action of its various components. In the future, it will be necessary to systematically evaluate this synergistic effect using experimental methods such as component separation and isopleth analysis, thereby providing further evidence to support its development as a new drug for the treatment of MAFLD.

Due to the multi-component nature of ARE, network pharmacology methods were employed to identify 137 common drug-disease targets. KEGG and GO analyses revealed enrichment in the lipid metabolism pathways. Several cancer-related pathways emerged in the enrichment analysis, which may be because MAFLD-associated hepatocellular carcinoma shares many of the same signaling pathways in its early stages. Furthermore, dysregulation of lipid metabolism is a critical biological process in tumor initiation and progression. Using Cytoscape (version 3.10.4, Cytoscape Consortium, San Diego, CA, USA), we then constructed a component–target–pathway network, and through topological analysis based on degree values, we identified ten core targets (AKR1B10, MMP2, EGFR, BACE1, HSD11B1, MAPK14, ERBB2, MET, STAT3, and APP) and ten core bioactive compounds (isorupestonic acid, rupestonic acid, quercetin, 6-[4-[2-carboxy-2-[(E)-3-(3,4-dihydroxyphenyl)prop-2-enoyl]oxyethyl]-2-hydroxyphenoxy]-3,4,5-trihydroxyoxane-2-carboxylic acid, linolenelaidic acid, caffeic acid, aurantiamide acetate and borneol 7-O.-[β-D-apiofuranosyl-(1→6)]-β-D-glucopyranoside (Ophiopogonin D), aurantiamide, and N-trans-feruloyltyramine. These compounds connected the greatest number of targets and pathways within the network, and from a network pharmacology perspective, represent the molecules with the greatest potential pharmacological contribution.

Consequently, our definition of ‘active compounds’ is not based on traditional pharmacokinetic parameters, but rather on target predictability and network topological importance. This screening strategy aligns with the ‘multi-component, multi-target’ research paradigm of network pharmacology, aiming to prioritize the key components with the highest connectivity within the network. Further experimental studies are required to determine the in vivo exposure levels of these core components, in order to clarify their pharmacokinetic characteristics.

Molecular docking was employed to verify the binding energies of the core targets to active constituents. The results indicated favorable binding affinity. AKR1B10, EGFR, HSD11B1, and STAT3 are all core targets that play important roles in lipid metabolism [26,27,28,29]. This suggests that the active constituents in ARE may regulate lipid metabolism disorders in MAFLD via a multi-component, multi-target, synergistic mechanism. Furthermore, several constituents (isorupestonic acid, rupestonic acid, aurantiamide acetate and aurantiamide) exhibited low binding energies to MAPK14 (<−9 kcal/mol), indicating a strong binding affinity. As MAPK14 plays a crucial role in regulating the activation of the inflammasome 3, this suggests that ARE may be valuable in inhibiting inflammatory infiltration during MAFLD progression [30]. For subsequent molecular dynamics (MD) simulation, we prioritized AKR1B10 over MAPK14. This choice was based on network topology rather than docking scores alone: AKR1B10 not only ranked first in degree centrality (Table A3), but also has been increasingly recognized as a regulator of de novo lipogenesis, whereas MAPK14, despite its stronger docking affinity, is a relatively non-specific inflammatory mediator. Therefore, considering the network ranking and the functional importance of AKR1B10 in MAFLD, together with its effective binding to characteristic compounds, we selected the top-ranked target AKR1B10, which exhibited binding energies of −6.6 and −6.7 kcal/mol with the characteristic compounds rupestonic acid and isorupestonic acid in ARE, respectively. Both values were lower than the activity threshold of −5 kcal/mol, suggesting that AKR1B10 possesses a strong binding affinity for these two compounds. We subsequently conducted molecular dynamics simulations to verify the binding stability of these two compounds with AKR1B10. The results indicated that isorupestonic acid maintained stable binding throughout the 100 ns simulation. Although this time span is sufficient for a preliminary assessment of binding stability and has been widely adopted in similar network pharmacology-guided molecular dynamics studies [31], it will be necessary in the future to conduct multiple independent replicate simulations to further enhance the statistical reliability of the binding analysis. Existing research indicates that AKR1B10 is highly expressed in livers affected by MAFLD. By regulating the protein levels of the PPAR signaling pathway, AKR1B10 influences the glucose and lipid metabolic pathways, thereby exacerbating disease progression [28]. Consequently, it is widely recognized as a reliable pharmacological target. Our results also demonstrated that ARE treatment significantly inhibits both the mRNA and protein expression of AKR1B10. These findings provide a solid foundation for the anti-MAFLD effects of ARE active components that target AKR1B10 and offer a theoretical basis for developing isorupestonic acid as a new, targeted anti-MAFLD drug candidate. In summary, our results suggest that the predicted ARE active components may have therapeutic potential for MAFLD via multiple mechanisms. Naturally, these findings require further experimental validation.

The primary pathological feature of MAFLD is a disturbance in hepatic lipid metabolism. This leads to the accumulation of lipids within hepatocytes, causing lipotoxic damage to these cells. If left unchecked, this results in hepatic inflammation and fibrosis. Therefore, the accumulation of lipids within hepatocytes is the initiating factor of this disease. In this study, we created a model of hepatic lipid deposition using oleic acid and assessed the potential impact of ARE on hepatocyte viability. The results showed that at concentrations ranging from 6.25 to 200 μg/mL, there was no significant impact on cell viability. Furthermore, Oil Red O staining and lipid content analysis confirmed that ARE inhibited lipid deposition in hepatocytes. Additionally, AST and ALT level measurements revealed that ARE exhibited hepatoprotective properties. These findings suggest that ARE may be effective in improving lipid accumulation in MAFLD. However, the in vivo efficacy of ARE still needs to be validated using a high-fat diet-induced MAFLD model characterized by inflammation and fibrosis. Further research should build upon these findings to validate the anti-MAFLD effects of ARE in animal models, elucidating the specific molecular mechanisms underlying its effect and providing stronger experimental evidence for the development of candidate therapeutic agents for MAFLD.

## 4. Materials and Methods

### 4.1. Reagents and Consumables

The dried aerial parts of *Artemisia rupestris* L. were purchased as herbal slices from Jiaheng Lengbei Pharmaceutical Co., Ltd. (Anguo, China; lot no. C202501001). The raw material was commercially sourced from Hotan (Xinjiang, China). A retained sample of the material has been deposited in our laboratory for future reference. The BCA protein assay kit (lot no. A045-3-2), low-density lipoprotein cholesterol (LDL-C) assay kit (lot no. A113-1), triglyceride (TG) assay kit (lot no. A110-1), high-density lipoprotein cholesterol (HDL-C) assay kit (lot no. A112-1), total cholesterol (T-CHO) assay kit (lot no. A111-1), alanine aminotransferase (ALT/GPT) assay kit (lot no. C009-2), and aspartate aminotransferase (AST/GOT) assay kit (lot no. C010-2) were all purchased from Nanjing Jiancheng Bioengineering Institute (Nanjing, China).

Paraformaldehyde (lot No. 80096618), sodium chloride (lot no. 10019318), sodium dihydrogen phosphate dihydrate (lot no. 20040718), disodium hydrogen phosphate dodecahydrate (lot no. 10020318), Oil Red O (lot no. 71029781, Walkey brand), isopropanol (lot no. 80109218), potassium aluminum sulfate (lot no. 10001060), glacial acetic acid (lot no. 10000218), glycerol (lot no. 10010618), and gelatin (lot no. 10010328) were obtained from Sinopharm Chemical Reagent Co., Ltd. (Shanghai, China). Hematoxylin (lot no. H9627-25G) was purchased from Sigma (St. Louis, MO, USA). Formic acid (chromatographic grade, lot no. 244885) was purchased from Merck (Darmstadt, Germany), acetonitrile (mass spectrometry grade, lot no. 24H2262019) from J.T. Baker (New Jersey, NJ, USA), and methanol (chromatographic grade, lot no. F25P5D205) from Fisher (Waltham, MA, USA). PBS solution was prepared in the laboratory using sodium chloride, sodium dihydrogen phosphate dihydrate, and disodium hydrogen phosphate dodecahydrate according to standard protocols. Oil Red O staining solution was prepared using isopropanol as the solvent, and hematoxylin staining solution was prepared with hematoxylin, potassium aluminum sulfate, glacial acetic acid, glycerol, and gelatin following standard procedures. All other conventional consumables and experimental water were of analytical grade.

HepG2 cells were purchased from Yousibo Biotechnology Co., Ltd. (Wuhan, China; lot no. YLH370). Sodium oleate (lot no. HY-N1446B) and atorvastatin (HY-B0589) were purchased from MedChemExpress (MCE, Monmouth Junction, NJ, USA). Anti-GAPDH (#10494-1-AP, 1:10000) and anti-AKR1B10 (#18252-1-AP, 1:1000) were purchased from ProteinTech (Wuhan, China).

KQ-300DE ultrasonic cleaner (Kunshan Ultrasonic Instrument Co., Ltd., Kunshan, China); RV8 rotary evaporator (IKA (Guangzhou) Instrument Equipment Co., Ltd., Guangzhou, China); Alpha 1-2 LD plus freeze dryer (Martin Christ, Osterode am Harz, Germany); refrigerated high-speed centrifuge (Thermo Fisher Scientific, Waltham, MA, USA); UPLC-Q-TOF-MS system consisting of an ACQUITY UPLC I-Class chromatograph and Xevo G2-XS QTOF mass spectrometer (Waters, Milford, MA, USA); Heracell 150i CO_2_ incubator (Thermo Fisher Scientific, Waltham, MA, USA); Class II A2 biological safety cabinet MSC Advantage II A2 (Thermo Fisher Scientific, Waltham, MA, USA); Varioskan LUX microplate reader (Thermo Fisher Scientific, Waltham, MA, USA).

### 4.2. Preparation of ARE

To recover phytochemicals with a wide range of polarities—including flavonoids, phenolic acids, and terpenoids—from plant materials, we selected 70% ethanol as the extraction solvent. The specific method is as follows. The dried plant material (500 g) was soaked in 2 L of 70% ethanol for 12 h, followed by ultrasonic extraction for 30 min. The resulting solution was centrifuged and filtered. The filtrate was then evaporated under reduced pressure using a rotary evaporator to remove ethanol, and subsequently lyophilized to obtain the total extract of *Artemisia rupestris* L. The extraction was performed in triplicate, yielding 26.7744 g, 27.5825 g, and 28.3990 g of lyophilized extract, respectively. The average extraction yield was 5.52% (*w*/*w*), calculated as (weight of lyophilized extract/weight of dried plant material) × 100%.

### 4.3. Chemical Composition Analysis of ARE

An aliquot of 100 mg of *Artemisia rupestris* extract was dissolved in an appropriate volume of methanol to achieve complete dissolution, followed by centrifugation at 12,000 rpm for 1 min. The supernatant was collected, filtered through a 0.22 μm microporous membrane, and transferred into an autosampler vial for subsequent analysis. Chromatographic separation was performed on a Waters ACQUITY UPLC HSS T3 column (2.1 mm × 100 mm, 1.8 μm, LC-226) maintained at 35 °C. The mobile phases consisted of (A) 0.1% formic acid in water and (B) 0.1% formic acid in acetonitrile. The flow rate was 0.3 mL/min, and the injection volume was 2 μL. Full-wavelength scanning was employed for detection. Mass spectrometry was conducted using an electrospray ionization (ESI) source with spray voltages set at 4 kV (positive ion mode) and 3.5 kV (negative ion mode). The sheath gas temperature was 325 °C at a flow rate of 8 L/min, and the drying gas temperature was 350 °C at a flow rate of 11 L/min. Data acquisition was performed in full-scan mode over a mass range of *m/z* 100–1700, with a cone voltage of 100 V and collision energy ramped at 10, 20, and 40 eV.

The raw data were imported into Qualitative Analysis 10.0 software, and an unknown compound identification workflow was established following the built-in wizards and method templates. Peak extraction was performed on the raw data. Putative molecular formulas were deduced based on the extracted molecular ion chromatograms and isotopic patterns of characteristic peaks. The MS/MS fragments were then matched against the PCDL tandem mass spectrometry database as well as an in-house *Artemisia rupestris* L. phytochemical database. Results with a mass deviation greater than 7.5 ppm or a matching score below 80 were filtered out. In parallel, the online database SIRIUS (version 5.8.5) was used to match the MS/MS fragments of characteristic peaks, retaining compounds with a matching score >70 and a FingerID score < 50. Ultimately, the chemical components in the *Artemisia rupestris* L. extract were successfully identified.

### 4.4. Bioinformatics Analysis

#### 4.4.1. Target Acquisition

Acquisition of MAFLD targets: The GEO database was searched using the keywords “MAFLD” and “NAFLD”, and five datasets were retrieved (Table A1). Differential expression analysis was performed on GSE89632, GSE160016, GSE126848, and GSE151158 using GEO2R. The union of the differentially expressed genes (DEGs) was then annotated using the GSE135251 dataset. The annotated genes were subjected to weighted gene co-expression network analysis (WGCNA). Specifically, the median absolute deviation (MAD) was calculated for each gene, and the top 50% of genes with the smallest MAD values were excluded. The goodSamplesGenes function from the R software package was subsequently applied to remove outlier genes and samples. A weighted co-expression network was then constructed. Genes belonging to the modules most significantly correlated with the clinical phenotype were defined as the MAFLD disease target set. The union-based DEG approach was chosen to maximize sensitivity and avoid missing potentially relevant genes due to heterogeneity among the four GEO datasets. The subsequent WGCNA module selection served as the primary filtering step to reduce the initial DEG pool to those most significantly correlated with the clinical phenotype.

#### 4.4.2. Acquisition of ARE Targets

The structural formulas of the 78 previously identified chemical components from the *Artemisia rupestris* L. extract were retrieved from PubChem. These structures were uploaded to the Swiss Target Prediction database for target prediction. During the initial screening phase, a threshold of “probability > 0” was applied based on existing network pharmacology studies to maximize the coverage of potential targets and minimize false negatives [32]. Ultimately, 74 compounds with predictable targets were retained, as some compounds either had no predicted targets or their structures could not be identified in the database. After deduplication, the drug target set was obtained. Finally, the intersection of the disease target set and the drug target set was taken to generate the set of potential targets for the treatment of MAFLD by *Artemisia rupestris* L.

#### 4.4.3. PPI Network and Enrichment Analysis

The set of potential targets was uploaded to the STRING database with the species set as Homo sapiens. The interaction score threshold was set to a medium confidence level of 0.4, and isolated nodes without any connections were hidden to generate the protein–protein interaction (PPI) network. Subsequently, the potential target set was submitted to the Meta scape platform, with the species also specified as Homo sapiens. The core parameters were configured as follows: minimum overlap threshold ≥ 3, significance level < 0.05, and enrichment fold > 1.5. Gene Ontology (GO) and Kyoto Encyclopedia of Genes and Genomes (KEGG) enrichment analyses were then performed.

#### 4.4.4. Construction of the Component–Target–Pathway Network

A component–target–pathway network was constructed using Cytoscape software. Topological parameters of the network were calculated using the built-in network analysis tool. Hub components and hub targets were identified based on their degree values.

#### 4.4.5. Molecular Docking

First, the top 10 core targets with the highest degree values were selected as receptor proteins. Their three-dimensional structures were retrieved from the PDB database. Water molecules and non-interacting residues were removed using the PyMOL Molecular Graphics System (version 2.5.4, Schrödinger, LLC, New York, NY, USA) and hydrogen atoms were added using AutoDock (version 4.2.6, Molecular Graphics Laboratory, The Scripps Research Institute, La Jolla, CA, USA). The processed structures were finally saved in .pdbqt format. Second, the molecular structure files of the top 10 core components with the highest degree values were downloaded from the PubChem database, processed with AutoDock (version 4.2.6, Molecular Graphics Laboratory, The Scripps Research Institute, La Jolla, CA, USA) software, and also saved as .pdbqt files. Finally, the AutoDock Vina (version 1.2.5, Molecular Graphics Laboratory, The Scripps Research Institute, La Jolla, CA, USA) plugin was used to calculate the binding free energy between each protein receptor and key active component to evaluate their affinity. The docking poses with the highest ranking were visualized in three dimensions using PyMol. Next, according to established convention, a binding energy threshold of −5.0 kcal/mol was used as the threshold for favorable interactions. This threshold is widely used in structure-based virtual screening to distinguish potential binders from non-binders [33]. To validate the docking protocol, we performed a redocking (self-docking) validation using AutoDock Vina (version 1.2.5, Molecular Graphics Laboratory, The Scripps Research Institute, La Jolla, CA, USA). The co-crystallized ligands—including EDO, NAP, and two 1WX molecules—from the target protein structure (PDB ID: 5LIW, single chain) were extracted and re-docked into the same binding pocket using the identical docking parameters described above. The RMSD values between the re-docked poses and the crystallographic conformations were 0.391 Å (EDO), 0.717 Å (NAP), and 0.91 Å (both 1WX ligands), all well below the commonly accepted threshold of 2.0 Å.

#### 4.4.6. Molecular Dynamics Simulation

The protein–ligand complex obtained from AutoDock Vina (version 1.2.5, Molecular Graphics Laboratory, The Scripps Research Institute, La Jolla, CA, USA) docking was separated into protein and ligand using the PyMOL Molecular Graphics System (version 2.5.4, Schrödinger, LLC, New York, NY, USA), and saved as Protein.pdb and ligand.mol2, respectively. The protein was refined in SPDBV (version 4.1.0, Swiss Institute of Bioinformatics, Lausanne, Switzerland), and the ligand was hydrogenated in Avogadro (version 1.2.0, Open Chemistry Project, Clifton Park, NY, USA) with its residue name changed to LIG. Ligand force field parameters were generated via CHARMM-GUI (Lehigh University, Bethlehem, PA, USA) Ligand Reader & Modeler (CGenFF). The solvated system was constructed using CHARMM-GUI (Lehigh University, Bethlehem, PA, USA) Solution Builder with NaCl ions, CHARMM36m force field, and GROMACS (version 2022.5, GROMACS Development Team, Stockholm, Sweden) format. Energy minimization, equilibration, and a 100-ns production run were performed using GROMACS (version 2022.5, GROMACS Development Team, Stockholm, Sweden) with GPU acceleration. Trajectories were processed for RMSD, RMSF, radius of gyration (Rg), SASA, and hydrogen-bond analyses. Free energy landscapes were constructed from RMSD and Rg via gmx sham. Binding free energies were estimated by MMPBSA using APBS (version 3.4.1, Pacific Northwest National Laboratory, Richland, WA, USA) over the last 10 ns, with energy components converted to kcal/mol and visualized in Origin (version 2024, OriginLab Corporation, Northampton, MA, USA). The complex was considered to have reached equilibrium when the RMSD values showed no significant upward drift and remained stable for the majority of the trajectory. Based on our RMSD analysis, the system reached equilibrium after approximately 20 ns of the 100 ns simulation. Therefore, the first 20 ns were excluded as the equilibration phase from downstream energetic analyses. All other analyses (RMSD, RMSF, Rg, SASA, and hydrogen bond) were performed using the full trajectory with the equilibration phase excluded.

### 4.5. In Vitro Cell Experiments

#### 4.5.1. Cell Model Establishment and Drug Treatment

HepG2 cells were cultured in Dulbecco‘s modified Eagle’s medium (DMEM) supplemented with 10% fetal bovine serum (FBS) and 1% penicillin–streptomycin at 37 °C in a 5% CO_2_ humidified atmosphere. To establish the MAFLD cell model, cells were seeded in appropriate culture plates, and after overnight attachment, treated with 0.5 mM SOA (dissolved in phosphate-buffered saline containing 10% bovine serum albumin) for 24 h. Cells cultured with an equivalent volume of vehicle served as the control group. After model establishment, cells were treated with 25, 50, and 100 μg/mL of the ARE for the indicated durations, and 10 μM atorvastatin was used as a positive control to validate the effectiveness of the experimental system [34].

#### 4.5.2. CCK-8 Assay

Cell viability was evaluated using the Cell Counting Kit-8 (CCK-8) assay (Dojindo Laboratories, Kumamoto, Japan). HepG2 cells were seeded in 96-well plates at a density of 5 × 10^3^ cells/well and subjected to the aforementioned treatments. After treatment, 10 μL of CCK-8 solution was added to each well, and the plates were incubated at 37 °C for 4 h. The absorbance was measured at 450 nm using a microplate reader. Cell viability was calculated as a percentage relative to the untreated control group.

#### 4.5.3. Cellular TG and TC Measurement

Intracellular TG and TC levels were determined using commercially available assay kits according to the manufacturer‘s instructions. Briefly, treated cells were harvested, washed twice with PBS, and lysed in cell lysis buffer. After centrifugation at 12,000× *g* for 10 min at 4 °C, the supernatant was collected. The TG and TC contents were measured using the GPO-PAP method, and protein concentrations were determined using the BCA assay. The TG and TC levels were normalized to total protein content and expressed as mmol/g protein.

#### 4.5.4. Cellular HDL-C and LDL-C Measurement

HDL-C and LDL-C levels in the cell lysates were measured using corresponding assay kits following the double-reagent direct method provided by the manufacturer. Absorbance was recorded at the wavelength specified in the kit protocol. HDL-C and LDL-C concentrations were calculated based on standard curves and normalized to total protein content.

#### 4.5.5. Oil Red O Staining

Intracellular lipid accumulation was assessed by Oil Red O staining. Cells were seeded in 6-well plates and treated as described above. After removal of the culture medium, cells were washed twice with PBS and fixed with 4% paraformaldehyde for 30 min at room temperature. The fixed cells were then stained with freshly prepared Oil Red O working solution (0.5% in isopropanol, diluted with distilled water at a 3:2 ratio) for 30 min. After staining, cells were washed three times with PBS, and the stained lipid droplets were observed under an inverted light microscope. For quantitative analysis, the Oil Red O dye retained in the cells was extracted with 100% isopropanol, and the absorbance was measured at 520 nm.

#### 4.5.6. AST and ALT Activity Assay

The AST and ALT activities in the cell culture supernatants were measured using commercial assay kits according to the manufacturer‘s protocols (Reitman-Frankel method). The absorbance was recorded at 510 nm, and enzyme activities were calculated using standard curves. Results were expressed as U/L.

#### 4.5.7. RT-qPCR

RNA was extracted using the Trizol method, in accordance with the provided protocol. The purity of the RNA was assessed using a Nanodrop spectrophotometer (Nanodrop 2000, Thermo Fisher Scientific, Waltham, MA, USA). Samples with an A260/280 ratio between 1.8 and 2.1 were selected for cDNA synthesis. The qPCR mix amplified the cDNA, and target gene expression was relatively quantified using GAPDH as the internal reference. Primers are listed as show in Table 3.

#### 4.5.8. Western Blot

The proteins were extracted using RIPA lysis buffer and their concentrations were determined using the BCA assay. Next, 5× loading buffer was added to the protein samples, which were then denatured by heating at 100 °C for 10 min. SDS-PAGE gel electrophoresis was then used to separate the proteins, which were transferred to a 0.22 μm nitrocellulose membrane. The membrane was then incubated overnight at 4 °C with primary antibodies under continuous agitation. This was followed by a two-hour incubation at room temperature with horseradish peroxidase-conjugated secondary antibodies (diluted 1:5000) that were specific to the primary antibody’s host species. The protein bands were visualized using a chemiluminescence detection system and their intensities were analyzed quantitatively using ImageJ software (version 1.53t, National Institutes of Health, Bethesda, MD, USA). Protein expression levels were normalized to GAPDH.

### 4.6. Statistical Analysis

First, the normality of the data was tested. If the data followed a normal distribution, the Brown–Forsythe test was used to assess the homogeneity of variances; a paired t-test was used for paired data, and an unpaired t-test for unpaired data. For comparisons between multiple groups, one-way or two-way analysis of variance (ANOVA) was selected according to the experimental design, followed by pairwise comparisons using the Student–Newman–Keuls test or Bonferroni correction. Non-parametric tests were used for data that did not follow a normal distribution. A *p*-value of <0.05 was considered to indicate a statistically significant difference.

## 5. Conclusions

In conclusion, this study systematically characterized the chemical composition of ARE, and through an integrated network pharmacology, molecular docking, MD simulation, and in vitro approach, revealed its multi-component, multi-target, and multi-pathway mechanisms against MAFLD. Potential key targets AKR1B10 and MAPK14 were identified through computational analysis, along with the characteristic components isorupestonic acid and rupestonic acid, which were identified as candidate compounds with potential activity by network pharmacology. Molecular dynamics simulations supported the prediction that isorupestonic acid formed a stable complex with AKR1B10, establishing AKR1B10 as a promising target for drug development., while cell-based assays confirmed ARE’s lipid-lowering and hepatoprotective activities. These results provide a scientific foundation for the further development of ARE as a therapeutic agent, and future studies in animal models are warranted to validate its efficacy and elucidate detailed molecular events.

## Figures and Tables

**Table 1:** The compounds of ARE.

No.	RT (min)	Formula	Molecular Weight	*m*/*z*	Mass Error (ppm)	Product Ions	Score	Compound	CAS or PubChem CID
1	1.084	C_7_H_12_O_6_	192.064	191.057	3.41	(−)109.0296, 125.4280, 158.8468	88.59	Quinic acid	36413-60-2
2	1.600	C_7_H_11_NO_5_	189.064	188.057	1.61	(−)102.0561, 128.0355, 144.0669	100	2-acetamidopentanedioic acid	5817-08-3
3	1.728	C_10_H_13_N_5_O_4_	267.097	268.104	1.44	(+)136.0617, 145.0493, 228.9667	82.7	Adenosine	58-61-7
4	2.208	C_7_H_6_O_5_	170.022	169.015	0.47	(−)102.0561, 111.0090, 128.0356	84.51	Gallic acid	149-91-7
5	3.084	C_9_H_11_NO_2_	165.079	166.086	0.87	(+)103.0541, 120.0807, 138.0556	93.8	L-Phenylalanine	63-91-2
6	3.951	C_14_H_20_O_9_	332.111	350.145	1.58	(+)111.0443, 139.0391, 171.0654	90.25	Leonuriside A	121748-12-7
7	4.081	C_14_H_18_O_9_	330.095	329.088	−0.3	(−) 123.0454, 152.0117, 167.0353	85.65	Vanillic acid glucoside	32142-31-7
8	4.643	C_22_H_32_O_15_	536.174	535.167	−1.01	(−)249.0768, 327.1079, 415.1239	74.01	[(2S,3R,4S,5S,6R)-3,4,5-trihydroxy-6-(hydroxymethyl)oxan-2-yl] (1S,4aS,7aS)-7-(hydroxymethyl)-1-[(2S,3R,4S,5S,6R)-3,4,5-trihydroxy-6-(hydroxymethyl)oxan-2-yl]oxy-1,4a,5,7a-tetrahydrocyclopenta[c]pyran-4-carboxylate	101831544
9	5.542	C_16_H_18_O_9_	354.095	355.103	0.98	(+)117.0336, 135.0443, 163.0394 (−)107.0501, 135.0453, 191.0563	91.46	1-O-Caffeoylquinic acid	1241-87-8
10	6.000	C_14_H_20_O_8_	316.116	317.124	1.37	(+)113.0596, 167.0700, 193.0854 (−)111.0455, 151.0767, 195.0659	83.21	Grevilloside G	791846-69-0
11	6.361	C_27_H_30_O_16_	610.153	611.161	−0.51	(+)187.0553, 449.1075, 502.1463	91.29	Kaempferol 3,7-di-O-glucoside	25615-14-9
12	6.642	C_7_H_6_O_3_	138.032	137.025	0.53	(−)109.0296, 119.0143, 108.0211	87.64	Protocatechualdehyde	139-85-5
13	6.689	C_15_H_16_O_9_	340.080	339.072	0.36	(+)105.0387, 179.0341, 151.0387 (−)177.0192, 228.9989, 215.9842	94.87	Cichoriin	531-58-8
14	8.326	C_15_H_16_O_8_	324.085	325.092	1.37	(+)163.0397, 187.1095, 257.0657	98.01	Skimmin	93-39-0
15	8.715	C_16_H_18_O_9_	354.096	355.104	2.99	(+) 135.0442, 145.0286, 163.0393 (−)127.0401, 173.0457, 191.0564	91.43	Cryptochlorogenic acid	905-99-7
16	8.886	C_15_H_14_O_6_	290.079	289.072	0.32	(−)245.0856, 227.0726, 230.0611	81.31	Catechin	154-23-4
17	9.684	C_16_H_18_O_9_	354.096	355.103	2.47	(+)291.0854, 207.0659, 237.0766 (−)111.0449, 135.0451, 191.0561	91.36	Chlorogenic acid	327-97-9
18	9.971	C_10_H_12_O_4_	196.074	197.081	0.5	(+)123.0440, 151.0394, 151.0389	98.12	Ethyl 4-hydroxy-3-methoxybenzoate	617-05-0
19	10.300	C_9_H_8_O_4_	180.042	179.035	0.35	(−)107.0502, 134.0370, 135.0453	91.76	Caffeic acid	331-39-5
20	10.773	C_18_H_28_O_9_	388.173	387.166	−1.03	(+)107.0852, 131.0856, 149.0964 (−)106.9957, 135.0459, 152.9365	82.41	2-[(1R,2R)-3-oxo-2-[(Z)-5-[3,4,5-trihydroxy-6-(hydroxymethyl)oxan-2-yl]oxypent-2-enyl]cyclopentyl]acetic acid	124649-25-8
21	11.174	C_21_H_20_O_11_	448.101	449.108	0.69	(+)181.0499, 231.0663, 287.0555	98.69	Luteolin-4-O-glucoside	6920-38-3
22	11.480	C_27_H_32_O_15_	596.174	595.166	−1.12	(−)119.0506, 235.0252, 355.0815	79.49	Viscumneoside III	118985-27-6
23	11.991	C_18_H_28_O_9_	388.173	406.208	2.36	(+)107.0855, 149.0962, 191.1069	89.85	β-D-Glucopyranosyl-11-hydroxyjasmonic acid	131752770
24	12.321	C_16_H_18_O_8_	338.101	339.108	1.06	(+)119.0492, 147.0443, 120.0253 (−)185.0268, 191.0557, 243.0524	99.01	5-O-(E)-p-Coumaroylquinic acid	5746-55-4
25	12.650	C_18_H_19_N_2_O_9_P	438.083	439.091	0.04	(+)147.0442, 175.0756, 203.0705 (−)125.0244, 167.0351, 237.0770	71.88	[(2R,3S,4S,5R)-5-(2,4-dioxo-5-prop-1-ynylpyrimidin-1-yl)-3,4-dihydroxyoxolan-2-yl]methyl phenyl hydrogen phosphate	5272832
26	13.256	C_16_H_20_O_10_	372.106	390.140	1.02	(+)105.0337, 179.0708, 337.0919	93.48	(2S,3S,4S,5R,6R)-6-(3-benzoyloxy-2-hydroxypropoxy)-3,4,5-trihydroxyoxane-2-carboxylic acid	45783079
27	14.036	C_25_H_24_O_12_	516.126	515.119	−0.65	(−)135.0451, 179.0348, 191.0563	95.43	1,4-Dicaffeoylquinic acid	1182-34-9
28	14.280	C_17_H_20_O_9_	368.111	369.119	1.16	(+)134.0364, 145.0285, 177.0549 (−)117.0348, 134.0376, 191.0563	94.96	4-O-Feruloylquinic acid	2613-86-7
29	14.794	C_27_H_24_O_18_	636.096	635.089	−0.45	(−)169.0145, 271.0457, 483.0779	87.37	1,3,6-Tri-O-Galloyl-β-D-Glucose	18483-17-5
30	15.656	C_15_H_20_O_3_	248.142	249.149	1.65	(+)147.0806, 175.1118, 189.1278	99.17	Rupestonic acid	83161-56-2
31	15.820	C_11_H_16_O_3_	196.110	197.118	1.48	(+)107.0855, 133.1014, 179.1071	78.82	Loliolide	5989-02-6
32	16.890	C_26_H_28_O_14_	564.148	565.156	0.62	(+)337.0715, 379.0815, 445.0937	94.42	Vicenin-1	35927-38-9
33	17.396	C_22_H_22_O_11_	462.116	461.109	0.67	(+)283.0598, 313.0707, 343.0810 (−)298.0487, 313.0353, 341.0679	97.02	Isoscoparin	20013-23-4
34	17.621	C_22_H_32_O_14_	520.179	521.188	1.76	(+)263.0918, 383.1342, 485.1660	71.75	1-[2-hydroxy-4,6-bis[[3,4,5-trihydroxy-6-(hydroxymethyl)oxan-2-yl]oxy]phenyl]-2-methylpropan-1-one	24990278
35	18.176	C_21_H_22_O_11_	450.116	451.124	1.3	(+)127.0390, 163.0390, 325.0920 (−)161.0244, 218.0224, 289.0696	72.94	3,4,2′,4′,6′-Pentahydroxychalcone 2′-glucoside	20126-63-0
36	18.843	C_20_H_22_O_6_	358.142	359.149	0.66	(+)137.0597, 175.0765, 191.0718	76.56	Dehydrodiconiferylalcohol	4263-87-0
37	19.507	C_14_H_6_O_8_	302.006	300.999	-0.15	(−)145.0296, 173.0243, 229.0147	83.2	Ellagic acid	476-66-4
38	19.781	C_27_H_30_O_16_	610.153	611.161	0.3	(+)181.0504 223.0802, 303.0504 (−)178.9993 271.0250, 300.0277	94.53	Rutin	153-18-4
39	20.123	C_22_H_32_O_14_	520.179	521.187	0.07	(+)181.0501, 317.1021, 353.1233	71.75	1-[2-hydroxy-4,6-bis[[(2S,3R,4S,5S,6R)-3,4,5-trihydroxy-6-(hydroxymethyl)oxan-2-yl]oxy]phenyl]-2-methylpropan-1-one	69178132
40	20.235	C_21_H_18_O_13_	478.075	479.082	0.38	(+)153.0183, 201.0552, 303.0501 (−)151.0041, 178.9987, 301.0357	87.13	Quercetin 3-O-D-glucuronide	22688-79-5
41	20.430	C_21_H_20_O_12_	464.096	465.104	1.58	(+)151.0037, 271.0248, 300.0276	99.14	Isoquercitrin	482-35-9
42	20.841	C_27_H_44_O_7_	480.309	481.316	1.11	(+)173.0960, 371.2232, 409.2753 (−)161.0246, 255.0300, 300.0277	79.92	Ecdysterone	5289-74-7
43	20.963	C_29_H_36_O_15_	624.204	623.197	−1.59	(−) 161.0243, 251.0533, 330.0324	97.37	Verbascoside	61276-17-3
44	21.268	C_25_H_24_O_12_	516.126	515.119	−1.01	(−)179.0350, 271.0953, 335.0797	92.7	Isochlorogenic acid B	14534-61-3
45	22.387	C_24_H_22_O_15_	550.097	551.1042	1.67	(+)127.0389, 229.0497, 303.0504	82.63	Quercetin-3-O-(6″-O-malonyl)-β-D-glucoside	96862-01-0
46	22.747	C_27_H_30_O_15_	594.158	593.151	−0.63	(−)255.0297, 285.0399, 366.0219	96.95	Luteolin-7-O-rutinoside	20633-84-5
47	23.342	C_25_H_24_O_12_	516.128	517.135	1.66	(+)135.0442, 145.0286, 163.0394	99.61	1,3-O-Dicaffeoylquinic acid	19870-46-3
48	23.583	C_24_H_24_O_14_	536.117	537.125	1.62	(+)127.0391, 163.0393, 411.0929 (−)16110246, 365.0872, 491.1194	74.41	6-[4-[2-carboxy-2-[(E)-3-(3,4-dihydroxyphenyl)prop-2-enoyl]oxyethyl]-2-hydroxyphenoxy]-3,4,5-trihydroxyoxane-2-carboxylic acid	131831523
49	23.577	C_21_H_20_O_11_	448.102	449.109	1.68	(+)213.0552, 241.0500, 287.0558 (−)135.1452, 191.0562, 353.0877	99.69	Kaempferol-7-O-glucoside	16290-07-6
50	23.850	C_26_H_28_O_15_	580.142	579.135	−1.69	(−)161.0243, 179.0355, 248.0631	92.71	Luteolin 7-primeveroside	44258133
51	24.413	C_22_H_22_O_12_	478.111	479.119	−1.59	(+)181.0494, 229.0494, 317.0658	99.3	Pedalitin glucoside	22860-72-6
52	25.045	C_20_H_18_O_10_	418.090	419.097	−0.35	(+)213.0551, 258.0525, 287.0553	87.31	Kaempferol 3-O-α-L-arabinopyranoside	99882-10-7
53	25.271	C_22_H_22_O_11_	462.116	463.124	−2	(+)258.0530286.0474, 301.0707	99.63	Swertiajaponin	6980-25-2
54	25.405	C_25_H_24_O_12_	516.127	515.120	0.49	(+)117.0335, 135.0442, 163.0394 (−)173.0458, 179.0351, 191.0562	95.29	4,5-Dicaffeoylquinic acid	57378-72-0
55	26.004	C_24_H_22_O_14_	534.101	535.110	1.95	(+)109.0283, 159.0290, 287.0555 (−)151.0043, 227.0350, 284.0326	91.84	Kaempferol 3-(6″-malonylglucoside)	81149-02-2
56	26.473	C_26_H_26_O_12_	530.142	531.150	1.83	(+)117.0335, 145.0287, 177.0550 (−)135.0457, 161.0250, 193.0516	97.86	3-Feruloyl-4-caffeoylquinic acid	96990-65-7
57	26.889	C_30_H_26_O_15_	626.128	627.135	0.78	(+)163.0394, 181.0196, 303.0503 (−)161.0247, 300.0278, 463.0880	95.58	Quercetin-3-O-(6″-O-Ecaffeoyl)-β-D-galactopyranoside	316354-12-8
58	27.177	C_26_H_26_O_12_	530.142	531.151	0.86	(+)117.0337, 145.0284, 177.0518 (−)173.0455, 335.0767, 367.1030	99.09	4-O-Feruloyl-5-O-caffeoylquinic acid	125132-81-2
59	28.349	C_34_H_30_O_15_	678.158	679.166	0.4	(+)135.0446, 145.0290, 163.0394 (−)191.0566, 353.0867, 394.0787	94.69	1,3,5-Tricaffeoylquinic acid	1073897-80-9
60	29.100	C_18_H_19_NO_4_	313.131	314.139	1.33	(+)117.0335, 145.0286, 177.0547	84.81	N-trans-Feruloyltyramine	66648-43-9
61 ^1^	29.519	C_34_H_30_O_15_	678.159	679.166	0.64	(+)145.0282, 163.0390, 337.0912 (−) 179.0353, 255.0665, 353.0880	93.9	Isomer of 1,3,5-Tricaffeoylquinic acid1	/
62	30.129	C_30_H_26_O_14_	610.132	611.140	0.57	(+)163.0392, 287.0552, 325.0919 (−)161.0246, 285.0405, 447.0932	95.58	Helichrysoside	56343-26-1
63	31.287	C_15_H_10_O_7_	302.043	303.050	1.3	(+)153.0183, 201.0550, 229.0496	98.88	Quercetin	117-39-5
64	31.293	C_21_H_36_O_10_	448.231	466.265	3.05	(+)115.0393, 137.1327, 145.0503	82.63	1-Borneol-β-apisyl-β-glucopyranoside	88700-35-0
65	31.722	C_28_H_32_O_14_	592.179	593.187	0.24	(+)163.0390, 285.0760, 447.1286	94.51	Buddleoside	480-36-4
66	39.500	C_18_H_34_O_5_	330.241	348.275	1.27	(+)109.1003, 155.1066, 227.2158 (−)127.1130, 171.1027, 211.1337	98.33	9,12,13-Trihydroxy-10E-octadecenoic acid	29907-56-0
67	41.508	C_59_H_92_O_27_	1232.583	1231.577	0.99	(−)1039.5118, 1099.5331, 1171.5547	76.28	(3β,4α,16α,21β,22α)-22-(Acetyloxy)-16,23,28-trihydroxy-21-[[(2Z)-2-methyl-1-oxo-2-buten-1-yl]oxy]olean-12-en-3-yl O-β-D-galactopyranosyl-(1→2)-O-[O-β-D-xylopyranosyl-(1→2)-α-L-arabinopyranosyl-(1→3)]-β-D-glucopyranosiduronic acid	131751749
68	43.820	C_25_H_26_N_2_O_3_	402.195	403.202	1.16	(+)105.0334, 152.1070, 224.1072	89.54	Aurantiamide	58115-31-4
69	48.500	C_18_H_37_NO_3_	315.277	316.286	3.88	(+)102.1277, 280.2638, 298.2744	88.89	Dehydrophytosphingosine	3687-54-5
70	49.322	C_15_H_22_O_3_	250.157	251.165	2.12	(+)105.0698, 131.0885, 145.1000	98.53	Isorupestonic acid	139953-21-2
71	49.789	C_27_H_28_N_2_O_4_	444.2049	445.2138	3.26	(+)105.0335, 194.1180, 224.1074	97.37	Aurantiamide acetate	56121-42-7
72	50.304	C_18_H_39_NO_3_	317.293	318.301	2.41	(+)109.1010, 282.2795, 300.2901	99.3	Phytosphingosine	554-62-1
73	50.919	C_18_H_34_O_4_	314.246	313.238	−0.83	(−)127.1126, 201.1125, 252.9892	100	(12Z)-9,10-Dihydroxy-12-octadecenoic acid	125356-86-7
74	51.950	C_33_H_56_O_14_	676.367	694.401	−0.26	(+)163.0605, 261.2209, 353.2686	98.27	Gingerglycolipid A	145937-22-0
75	56.294	C_18_H_30_O_2_	278.225	279.232	3.88	107.0854, 109.1012, 173.1323	93.57	Elaidolinolenic acid	28290-79-1
76	57.277	C_18_H_30_O_3_	294.219	293.21	−0.95	(+)105.0699, 151.1123, 227.2164 (−)134.8934, 157.0276, 187.0030	71.47	13-Oxo-9E-11E-octadecadienoic acid	29623-29-8
77	64.273	C_35_H_36_N_4_O_6_	608.264	609.272	1.59	(+)503.2440, 531.2390, 550.2563	78.95	3,3′,3″-(12-Ethenyl-3,8,13,17-tetramethylporphyrin-2,7,18-triyl)tripropanoic acid	30783-27-8
78	65.840	C_34_H_40_O_9_	592.268	591.261	1.25	(+)460.2235, 505.2234, 533.2551 (−)255.2340, 338.1546, 515.2440	74.18	Isomoreollic acid	1240792-57-7

^1^ 61 is an isomer of 59.

**Table 2:** The correlation between MAFLD features and each module.

Module	Correlation (R)		Significance (p)	
	MAFLD	Control	MAFLD	Control
Grey	0.29	−0.29	1.5 × 10^−5^	1.5 × 10^−5^
Blue	0.20	−0.20	3.1 × 10^−3^	3.1 × 10^−3^
Green yellow	−0.08	0.08	0.24	0.24
Dark turquoise	−0.19	0.19	4.2 × 10^−3^	4.2 × 10^−3^
Dark green	0.26	−0.26	9.7 × 10^−5^	9.7 × 10^−5^
Light green	−0.39	0.39	2.9 × 10^−9^	2.9 × 10^−9^
Dark grey	0.25	−0.25	1.5 × 10^−4^	1.5 × 10^−4^
Black	1.7 × 10^−3^	−1.7 × 10^−3^	0.98	0.98
Cyan	−0.18	0.18	7.4 × 10^−3^	7.4 × 10^−3^
Dark orange	−0.02	0.02	0.72	0.72

**Table 3:** Primer sequences for quantitative real-time PCR.

Primer Name	Nucleotide Sequence (5′–3′)	Tm Value	CG%	Product Length
H-GAPDH	Sense CATCATCCCTGCCTCTACTGG	50.9	57.1	259
	Antisense GTGGGTGTCGCTGTTGAAGTC	60.1	57.1	
H-AKR1B10	Sense CATTGACTGTGCCTATGTCTATCAG	59.3	44	203
	Antisense TAAGATAGACGTCCAGATAGCTCAG	59.1	44	

## Data Availability

The original contributions presented in this study are included in the article. Further inquiries can be directed to the corresponding author.

## Abbreviations

The following abbreviations are used in this manuscript:

AKR1B10	Aldo-Keto Reductase Family 1 Member B10
ALT	Alanine Aminotransferase
APP	Amyloid Precursor Protein
ARE	*Artemisia rupestris* L. extract
AST	Aspartate Aminotransferase
BACE1	Beta-Secretase 1
DEGs	Differentially Expressed Genes
EGFR	Epidermal Growth Factor Receptor
ERBB2	Erb-B2 Receptor Tyrosine Kinase 2
HDL-C	High-Density Lipoprotein Cholesterol
HSD11B1	Hydroxysteroid 11-Beta Dehydrogenase 1
LDL-C	Low-Density Lipoprotein Cholesterol
MAFLD	Metabolic Dysfunction-Associated Fatty Liver Disease
MAPK14	Mitogen-Activated Protein Kinase 14
MD	Molecular Dynamics
MET	MET Proto-Oncogene, Receptor Tyrosine Kinase
MM/PBSA	Molecular Mechanics/Poisson-Boltzmann Surface Area
MMP2	Matrix Metallopeptidase 2
SOA	Sodium Oleic Acid
PPI	Protein–Protein Interaction
RMSD	Root-Mean-Square Deviation
RMSF	Root-Mean-Square Fluctuation
SASA	Solvent-Accessible Surface Area
STAT3	Signal Transducer and Activator of Transcription 3
TC	Total Cholesterol
TG	Triglyceride
UPLC-Q-TOF-MS	Ultra-Performance Liquid Chromatography-Quadrupole-Time-of-Flight-Mass Spectrometry

## Appendix A

**Table A1:** GEO accession and sample information.

Dataset ID	Sample Size	Sex		Age		Diagnosis	
		Male	Female	<60	≥60	Healthy	MAFLD
GSE89632	44	25	19	42	2	24	20
GSE160016	11	6	5	11	0	6	5
GSE135251	216	/	/	/	/	10	206
GSE126848	29	23	6	/	/	14	15
GSE151158	45	20	24	41	3	21	24

**Table A2:** The number of genes in each module.

No.	Module	Number of Genes
1	grey	2182
2	blue	958
3	green yellow	809
4	dark turquoise	400
5	dark green	221
6	light green	200
7	dark grey	174
8	black	146
9	cyan	100
10	dark orange	37

**Table A3:** Top targets and pathways ranked by degree centrality in the component–target–pathway network.

Rank	Degree	Name	Type
1	21	Isorupestonic acid	Drug
2	20	Rupestonic acid	Drug
3	19	Quercetin	Drug
4	18	6-[4-[2-carboxy-2-[(E)-3-(3,4-dihydroxyphenyl)prop-2-enoyl]oxyethyl]-2-hydroxyphenoxy]-3,4,5-trihydroxyoxane-2-carboxylic acid	Drug
5	16	Linolenelaidic acid	Drug
6	15	Aurantiamide acetate	Drug
7	14	Caffeic acid	Drug
8	14	Borneol 7-O-[β-D-apiofuranosyl -(1→6)]-β-D-glucopyranoside	Drug
9	13	Catechin	Drug
10	13	Aurantiamide	Drug
1	16	AKR1B10	Target
2	15	MMP2	Target
3	15	EGFR	Target
4	12	MAPK14	Target
5	12	HSD11B1	Target
6	12	BACE1	Target
7	11	MET	Target
8	11	ERBB2	Target
9	9	STAT3	Target
10	9	APP	Target
1	14	Lipid and atherosclerosis	Pathway
2	13	Chemical carcinogenesis—reactive oxygen species	Pathway
3	11	Apoptosis	Pathway
4	10	Focal adhesion	Pathway
5	10	Proteoglycans in cancer	Pathway
6	9	Th17 cell differentiation	Pathway
7	9	MAPK signaling pathway	Pathway
8	9	NOD-like receptor signaling pathway	Pathway
9	7	EGFR tyrosine kinase inhibitor resistance	Pathway
10	6	Central carbon metabolism in cancer	Pathway
